# Training to Transition: Using Simulation-Based Training to Improve Resident Physician Confidence in Hospital Discharges

**DOI:** 10.15766/mep_2374-8265.11348

**Published:** 2023-09-15

**Authors:** Jenna Sizemore, Andrea Bailey, Spoorthi Sankineni, Karen Clark, Shanthi Manivannan, Maria Kolar, Mary Warden, Sarah Sofka

**Affiliations:** 1 Assistant Professor and Associate Program Director, Internal Medicine Residency Program, Department of Medicine, West Virginia University School of Medicine; 2 Clinical Assistant Professor and Director of Simulation, West Virginia University School of Nursing; 3 Consulting Associate, Duke Primary Care and Department of Medicine, Duke University School of Medicine; 4 Professor, Department of Medicine, West Virginia University School of Medicine; 5 Associate Professor and Section Chief, Department of Medicine, West Virginia University School of Medicine; 6 Professor, Department of Medicine, and Associate Program Director, Transitional Year Residency Program, West Virginia University School of Medicine; 7 Associate Professor, Department of Medicine and Department of Medical Education, and Program Director, Transitional Year Residency Program, West Virginia University School of Medicine; 8 Professor, Department of Medicine, and Program Director, Internal Medicine Residency Program, West Virginia University School of Medicine

**Keywords:** Discharge, Handoff, Transition of Care, Continuity of Care, Hospital Medicine, Simulation, Standardized Patient

## Abstract

**Introduction:**

Hospital discharge is a highly critical and complex process that is prone to medical errors, poor communication, and ineffective synchronization of transitional teams. Improving safety during postacute care transitions has become a national focus. Simulation-based training is an underutilized method of instruction for medical resident transitions of care education.

**Methods:**

As an integral part of a transitions curriculum, 36 PGY 1 residents from internal medicine and transitional year residency programs underwent a discharge simulation utilizing a trained simulated participant (SP) and a lay caregiver. The objective of the training was to implement a simulation-based education intervention to improve transition practices and discharge communication in graduate medical education. A faculty observer used a case-specific discharge rubric to standardize feedback to the resident and observed the resident navigate the electronic medical record (EMR) for discharge orders. Pretest and posttest surveys assessing resident attitudes and confidence regarding specific areas of the discharge process were distributed to all participating residents for completion.

**Results:**

Thirty-six internal medicine and transitional year residents (100%) completed an observed discharge simulation with an SP and a separate encounter with the EMR discharge navigator. All 36 residents (100%) completed the pretest survey, and 23 (63%) completed the postsurvey evaluation. Postsurvey results showed residents agreed (92%, *p* < .05) that the simulation increased their confidence in safely discharging a patient.

**Discussion:**

Simulation encounters are an effective adjunct to postacute care transition education.

## Educational Objectives

By the end of this activity, learners will be able to:

1. Identify relevant details from a discharge summary comprising a brief patient history, hospital course, and medication list.
2. Execute patient discharge orders in an electronic medical record using a playground computer system.
3. Communicate accurate discharge orders about medications, outpatient appointments, and outpatient laboratory investigations to a patient and caregiver based on a discharge rubric with case-specific goals.
4. Respond effectively to patient and caregiver inquiries and concerns in a simulated discharge interaction.
5. Improve their confidence in performing hospital discharges.
6. Recognize the importance of utilizing a lay caregiver in performing hospital discharge.

## Introduction

Hospital discharges are fraught with medical errors, often stemming from medication changes, poor communication of the transition plan to patients and caregivers, and lack of coordination between the transitional teams.^1–3^ Patients with low health literacy are more vulnerable during the postacute care transition process.^4^ Lack of patient education on postacute needs, as well as medication changes, creates a scenario of rushed discharges with increased risk of errors.^1^ The Accreditation Council for Graduate Medical Education's common program requirements require resident physicians to coordinate patient care across all health care sectors and to demonstrate communication skills that promote the effective exchange of information with patients, families, and health professionals.^5^ Many residents learn discharge habits during active patient care, similar to the adage “See one, do one, teach one.”

Formal education regarding transitional medical errors and patient perspectives in discharge decision-making can be limited in residency training. Prior programs have noted success in using simulation to teach discharge practices, specifically within a pediatric setting.^6,7^ The complexity of performing a safe discharge with patients suffering from geographic health care disparities can be overwhelming to early learners, as well as increasing the risk of inadvertent medical errors. Hence, we focused on developing an educational intervention utilizing simulation to highlight complex discharges. Simulation has become a valuable adjunct to educational practices in graduate medical education.^8,9^ Designed to create deliberate and repetitive practice in an interactive environment, health care simulation has been effective for procedural training, communication, and electronic medical record (EMR) simulated exercises.^8–10^ Simulated participant (SP) encounters are widely used in medical education to develop communication skills with the benefit of direct learner-centered feedback.^9,10^ The use of medical simulation as an instructional modality has a background in Ericcson's theory of deliberate practice, wherein learners utilize guided practice activities to improve a specific aspect of their performance.^11,12^ As institutions develop transitions curricula within residency programs, SP encounters can be an effective method to teach safe transition practices to learners using the theory of deliberate practice.^11,12^

## Methods

### Development

An interdisciplinary team consisting of physicians and an advanced practice provider created a hospital discharge simulation, utilizing feedback and comments from licensed nurses and clinical pharmacists on the developed cases. Simulated patient encounters were initiated at the start of an academic year. Nine encounters were conducted in half days and longitudinally throughout the first 2 months of the academic curriculum, with four residents per session. Participants were PGY 1 residents from the internal medicine and transitional year residency programs at West Virginia University. Residents completed the intervention within the first 2 months of starting training. Simulated patient encounters were completed at the David and Jo Ann Shaw Center for Simulation Training and Education for Patient Safety.

We utilized Ericsson's foundational elements for deliberate practice, including motivation, well-defined objectives, measurable metrics of performance, focused practice skills, and real-time actionable feedback.^11,12^ A formal lecture (Appendix A) discussing components of a successful discharge, as well as a discharge checklist, was given prior to starting the simulation. The learning objectives listed in Appendix A differed from those utilized for the simulation to allow the lecture to be used in educational efforts separate from the simulation scenario. A prebrief (Appendix B) that included goals of the simulated encounter was given to all participants. Two scenarios depicting a patient on the day of hospital discharge—listing a brief patient history, hospital course, and a medication list—were reviewed by the residents. The patient scenarios were designed to highlighted discharging a patient with several comorbid conditions from an urban, academic medical center to a rural area. Case 1 (Appendix C) highlighted a discharge with a new diagnosis of diabetes mellitus type II requiring insulin and coronary artery disease. Case 2 (Appendix D) described a patient with recent mechanical heart valve replacement requiring anticoagulation, as well as medication-assisted treatment for opioid use disorder (OUD). These cases were designed to improve the safety of discharges with potential for adverse medication events while emphasizing geographic health care disparities prevalent in rural medicine. We chose these cases to depict common scenarios within our patient population that had been anecdotally identified as often having discharge errors. Residents were randomized to either case due to SP availability portraying the developed cases.

After completion of the simulated patient part of the encounter, a PGY 1 resident learner placed discharge orders in a playground EMR using our institution's practice EMR environment. Orders were entered for discharge medications, outpatient appointments, and outpatient laboratory investigations. After learners had completed their discharge orders, an observing faculty described common pitfalls within the EMR and discussed utilizing a discharge checklist (contained in Appendix A) during the discharge process to ensure correct order placement. The discharge checklist was available within the EMR for the learner to reference during order entry. This checklist mirrored the same checklist reference tool that resident physicians utilized during active patient care.

The simulated sessions included an SP and a transitions team staff member as a participant in the role of lay caregiver. We utilized two separate SPs to allow for successful implementation of the scenario based on limited SP availability. SP education was provided by a member of the interdisciplinary team and consisted of roughly 1 hour of education with case review by the SP, as well as anticipatory guidance involving discussion items to be highlighted during the debriefing, focusing on communication practices from the learner. Feedback from the SP regarding the implementation of the encounter, as well debriefing feedback, was utilized throughout the encounter. The resident was unaware that the lay caregiver was a staff member. We intentionally created this model to impart a multidisciplinary approach to feedback, as our transition teams comprised advanced practice providers, registered nurses, medical assistants, and general internal medicine faculty. The lay caregiver assumed the role of a family member and was given a script of potential questions to ask the resident during the encounter, including medication and follow-up appointment clarification, as well as any potential concerns about the home transition. The SP also aided the resident in keying toward the objectives, if needed. A facilitator, either a faculty physician or an advanced practice provider, observed the resident perform the discharge behind a two-way mirror.

A discharge rubric with case-specific goals (Appendix E) was completed by an observing faculty member, as well as by the team member in the role of lay caregiver. Rubric categories included reason for hospitalization, symptomatic warning signs, discharge diet and activity level, wound care, discharge medication review, outpatient appointments, unique patient needs, lay caregiver questions, and medication obtainment. The rubric was developed by the transitions team, focusing on the most common errors its members had experienced in reviewing hospital discharges and following patients postdischarge within our respective hospital system. A pilot was conducted with the clinical members of the developing team, with input from SP, to determine timing and flow, as well as to ensure the rubric could be successfully used in the encounter; postgraduate learners were not utilized due to scheduling barriers. After completion of the pilot encounter, the facilitators met as a group to review scoring on the rubric, with input and consensus by the interdisciplinary team. Evaluators scored each category individually. The maximum points in each category varied based on complexity and ranged from 2 to 8. The resident learner was debriefed immediately after conclusion of the scenario, receiving feedback from the SP, the lay caregiver, and the observing faculty member using the discharge instructions rubric, with the feedback team and learner often reflecting on any barriers that may have arisen from social determinants of health. The debriefing usually consisted of using the plus-delta method, given the focus on learner self-assessment and reflection, as well as faculty familiarity with this debriefing method. The performance of the resident during the simulated encounter did not impact the educational standing in any way and was a low-stakes encounter centered on formative feedback to the learner. The rubric was used to guide discussion on discharge errors during the morbidity and mortality conference as a standard process in a safe setting, which had not been done previously. This was implemented after the development of this training and, given its nature, was not tracked in the aggregate.

Residents voluntarily completed a presimulation survey to assess baseline characteristics in self-reported confidence in the discharge process, perceptions of using a lay caregiver, and perceived responsibility of the discharging physician. The survey used a 5-point Likert scale (1 = *strongly disagree,* 5 = *strongly agree*). Positive responses were indicated by scores of 4 and 5. A postsimulation survey was distributed for voluntary completion after all PGY 1 participants had completed the simulation, roughly within 2–3 months of clinical training. Residents could make anonymous comments on the effectiveness of the simulation after completion, in addition to feedback, if they felt the patient scenario was helpful in learning safe discharge practices. The postsimulation survey included statements on the effectiveness of using an SP scenario in teaching safe discharge practices. Pre- and postsimulation surveys were different, as postsimulation data were collected at different time periods throughout the intern year due to simulation scheduling, after all PGY 1 residents had completed the survey. Due to anonymity concerns, the surveys were not linked in order to safeguard resident responses. Both surveys were designed by the team to assess learners’ perspective and confidence regarding discharge. Surveys were developed with respect to the cases because, to our knowledge, a validated survey was not available at the time of implementation. Program personnel used an online evaluation tool to collect all responses anonymously and in the aggregate. Two survey items were intentionally linked to assess efficacy of the simulation. Statistical analysis of the linked responses was performed using Fisher's exact test for count data.

### Equipment/Environment

The simulation used the following equipment and materials:

• Lecture materials
• Patient case scenarios, including a mock discharge summary
• Discharge instructions rubric/checklist
• Practice EMR environment
• Simulated hospital room
• Hospital bed
• Hospital gown for SP
• Noninvasive blood pressure cuff
• Stethoscope
• Peripherally inserted central venous catheter (attached to patient)

### Personnel

An SP was provided by the simulation center. The facilitator team performed all simulator training; a small number of simulators were trained for each specific case. As all facilitators were involved in the development phase of the simulation, no additional facilitator training was required. An SP lay caregiver, who was a member of the transitional care team, performed the role of a family member. The case facilitator served as an observer. All facilitators were physicians or advanced practice providers. The learners comprised PGY 1 residents from an internal medicine residency and transitional year residency program. Simulation center personnel escorted learners from a waiting room to the location of the simulated encounter.

### Implementation

The simulation was performed in our simulation center within standardized practice exam rooms. The simulation staff took the learners to the simulation room. Resident learners performed the encounter independently to develop autonomy in the learner as well to mirror a potential common practice within their respective clinical setting. The learners had already completed the prebrief (Appendix B) with a member of the facilitator team. The simulation staff, as well as the prebrief, told the participants that the setting was a general unmonitored hospital room. A facilitator was placed in a different room behind a two-way mirror to directly observe the simulation. The SP and lay caregiver were already in place within the exam room, and the simulated encounter started upon the learner's entry into the exam room. Direct interaction with the SP took roughly 20 minutes per learner for completion. Feedback from the observing facilitator, the lay caregiver, and the SP was allotted 10 minutes. Direct supervised placement of discharge orders into a practice EMR took an additional 10 minutes immediately following the SP encounter; this was performed outside the simulation room due to equipment constraints.

### Debriefing

The facilitator first asked the learner to summarize the simulation and to reflect on any potential postdischarge risks that might be applicable to the SP. The facilitator reviewed the learning objectives and asked the learner what had gone well and what they would do differently, if applicable, utilizing a plus-delta method of debriefing. They reviewed potential areas of discussion based on the case-specific rubric. The lay caregiver and SP reflected on communication practices. The learner's questions and feedback were elicited during this time. All learners were debriefed individually. We developed case-specific rubrics and debriefing materials (Appendix E) to standardize feedback across the facilitator team; a point system identified future potential education targets for discharge education but did not impact the learners’ academic standing or work performance. The plus-delta method of debriefing was most often utilized given the emphasis on self-assessment and was done in person to allow discussion and discourse. The facilitators had previously met as a group, with input from and consensus by the interdisciplinary team, to standardized scoring and use of the rubric. After completion of feedback, the learner practiced placement of discharge orders in the playground EMR under direct supervision. This supervision was performed by a faculty physician.

### Assessment

The facilitator reviewed general topics from the discharge checklist as well as items from the case-specific rubrics (Appendix E). Learners received formative feedback. The observing facilitator and the lay caregiver completed the rubric to guide feedback to the learner. Rubric scores were later collected anonymously and in the aggregate to identify future educational targets; individual scores were not displayed to residents. Aggregate scores and grouping were presented to all resident physicians and faculty members after 6 months of completing the scenarios. Resident physicians reflected on their experience of this simulation in a large-group setting, as well as on subsequent real-patient encounters that promoted discussion of the challenges in hospital discharges. The general grouping of the discharge rubric was available as a resource for morbidity and mortality conferences should the residents need to highlight any cases from postacute transition. A discharge checklist resource was created within our playground EMR as a resource for residents to reference when discharging a patient.

## Results

All participants (*N* = 36) completed the presimulation survey and participated in the complete discharge simulation. Before participating in the simulation, residents reported low confidence in safely discharging patients, with only 28% agreeing they felt confident in discharging a patient (Table 1). They reported ambivalence in their perceived ability to write clear discharge instructions for patients, with a median score of 3 (*neither agree nor disagree*.) The residents were able to recognize the importance of incorporating a lay caregiver in the discharge process, with 100% agreement, and they felt that the discharging physician was ultimately responsible for all the discharge needs (Table 1).

**Table 1.**
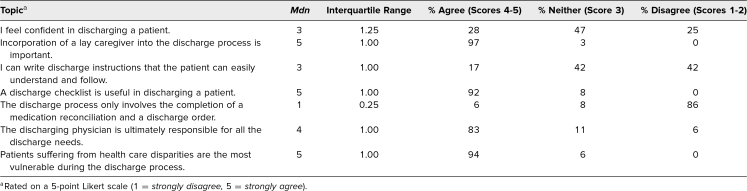
Presimulation Survey (*n* = 36, Response Rate = 100%)

Participants were randomly assigned to either case 1 (*n* = 14) or case 2 (*n* = 22) based on trained SP availability; distribution was unequal due to SP scheduling. Rubric scores for each case were similar; the average score for case 1 was 72% (*SD* = .084), and the average score for case 2 was 69% (*SD* = .088; Figure). Discussing medication obtainment in case 2 received the lowest score of all categories, with an average of 50%. The case utilized a discharge on buprenorphine/naloxone for medication-assisted treatment for OUD. In case 2, residents also received lower scores (*M* = 55%) when addressing the other unique needs of OUD treatment, which included community resources and access to behavioral medicine (Figure). In both cases 1 and 2, residents received 64% for addressing symptomatic warning signs, such as hypoglycemia in case 1, and discussing signs of bleeding and fevers in case 2. The participants received average scores of 57% and 59%, respectively, in the lay caregiver category of cases 1 and 2 (Figure).

**Figure.**
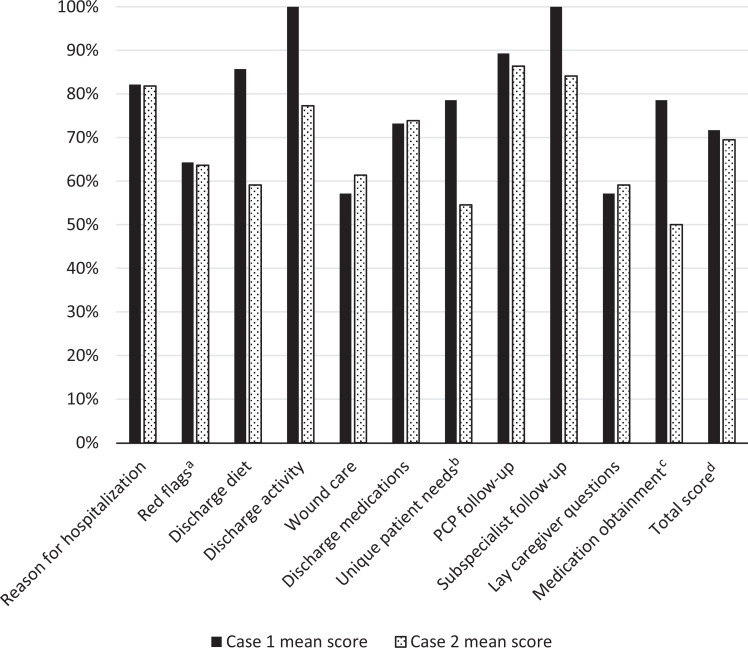
Simulation evaluations. The maximum points in each category varied based on complexity and ranged from 2 to 8. Points received in each category were later converted to percentages to standardize the scores. Scores were utilized to identify further educational targets in the residency curricula. ^a^Red flags for case 1 included discussing symptoms of hypoglycemia and hyperglycemia as well as chest pain; red flags for case 2 included the development of fevers, bleeding, and/or bruising, as well as signs of relapse. ^b^Unique patient needs for case 1 included need for home care; unique patient needs for case 2 included addressing access to outpatient behavioral medicine, available community resources, and transportation for laboratory monitoring. ^c^Medication obtainment for both cases included addressing medication affordability, need for any prior authorization, and possible need for written prescription. ^d^For the total score of case 1, *SD* = .084; for the total score of case 2, *SD* = .088.

Twenty-three residents responded to the postsimulation survey (63% response rate), which was given following completion of the simulation curriculum by all participants, after 2 months of residency training. After participating in the simulation, residents reported that their confidence in discharging a patient had dramatically increased, with 92% (*p* < .05) agreeing the simulation improved their confidence in discharging a patient (Table 2). Also, 83% of residents felt that they were more confident in using the EMR discharge navigator (Table 2.) As part of the survey, residents were asked to voluntarily comment on the strengths and weakness of the simulation. Several responded that they valued the direct feedback incorporated in the simulation. Weaknesses noted included time constraints and a desire for more information about system practices and community resources available to patients.

**Table 2.**
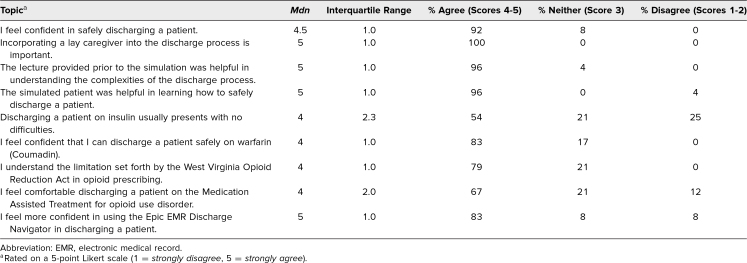
Postsimulation Survey (*n* = 23, Response Rate = 63%)

## Discussion

Our simulation-based educational intervention focused on creating a multifaceted approach to improving PGY 1 residents’ education on safe discharge practices from the hospital. Postacute care transition has been shown to be a problematic and time-consuming process that can leave patients perplexed.^13,14^ A prospective cohort study in 2003 showed that 20% of discharged patients had adverse events transitioning from the hospital to home, two-thirds of which could have been prevented or mitigated.^15^ Many adverse events that occur from discharge are rooted in systemic errors in the transitions process and hospital system, including medication errors, procedural complications, and nosocomial infections and injuries.^1–3,15^ There has been evidence to suggest benefit in team-based discharge planning, with effective communication practices between health care professions integral for patient safety.^16^ Our simulation was designed to highlight the importance of interprofessional communication and utilized an interdisciplinary approach to case development, implementation, and debriefing.

Resident physicians perceive that they do not receive formal training in transitions practices; instead, they commonly learn these processes in the form of peer-to-peer instruction while actively discharging their patients.^14^ Our simulation cases were specifically designed to target self-reported knowledge gaps among our residents. For example, resident physicians often have varying degrees of familiarity with the diagnosis and treatment of OUD; the case 2 scenario was designed to familiarize them with the challenges that occur from a lack of access to treatment, especially within a rural area. These case scenarios were designed to emphasize safety in discharges, while highlighting the health care disparities prevalent in rural medicine.

Residents are routinely expected to independently discharge patients throughout their training. However, the lack of dedicated training may contribute to the adverse events that occur with postacute care transitions. We developed our stimulation-based educational intervention to teach a standardized discharge process to our residents. The lectures and simulation experience expounded on the importance of safe discharges and the physician's role in this process. Many of the surveyed residents agreed that the discharging physician is ultimately responsible for all discharge needs. However, many also struggled with providing guidance on disease-specific red flags, unique patient needs, and wound care. This suggests a discordance between residents’ perceived responsibilities and their true discharge process. We additionally learned that several residents did not have dedicated discharge training during their undergraduate medical education. Limiting our analysis was the lower completion rate of the postsimulation survey due to its voluntary nature. In order maintain anonymity, we were unable to link completed pre/post survey responses given that several team members were integral to the residency program leadership. Another limitation was conducting this simulation within one cohort year. Ideally, we would have repeated the simulation with subsequent resident cohorts. Unfortunately, the COVID-19 pandemic limited our ability to perform in-person simulation events. The cases were modified into a virtual encounter focusing on a postdischarge follow-up appointment. While the intent of the educational objectives remained similar, the simulation ultimately differed enough that the rubrics and surveys could not be generalized to subsequent years.

The residents overwhelmingly agreed that incorporating the lay caregiver in the discharge process was important, yet they struggled with including the lay caregiver unless prompted. When residents had difficulty, the lay caregivers were permitted to prompt them with specific questions that patients routinely ask at discharge. Directed feedback was provided by advanced practice providers, nurses, and medical assistants, who were well versed in coordinating pre- and postdischarge needs; to preserve anonymity, these comments were not recorded as they were often individualized to the resident and frequently described the participants’ past personal experiences in health care. Residents’ evaluation comments noted that this feedback provided specific guidance on improving their discharge process and was a strength of the simulation encounter. Residents’ postsimulation comments, including specific comments on after-visit summary review and the helpfulness of the discharge checklist, also indicated that they were unaware of several processes in place to help facilitate safe discharges. Subsequently, the discharge checklist was offered as a reference within the system EMR for all staff to reference when discharging patients. Constructive comments from residents concerned the timing of performing this simulation encounter, as they felt it was most helpful 3 months into the PGY 1 year, to allow more comfort with the EMR and to more effectively assess their clinical growth after 3 months of residency training.

Traditionally, physicians have been expected to develop expertise in patients transitioning through experience and peer-to-peer education. Unfortunately, that approach can lead to a wide variation in skill development. It also has the potential to cause misinformation, medical errors, readmissions, and poor patient communication.^14,15^ While observing the simulated patient interactions, we recognized that our resident learners needed further education—on identifying disease-specific warning signs and unique patient needs and on relaying this information to patients and caretakers at discharge. Evaluation comments noted enhanced discharge skills postsimulation; our residents agreed that simulation was an effective tool in postacute care transition education. While initially created for this simulation, the discharge checklist taught during the discharge order entry developed into a clinical tool available with system EMR as a reference for residents to use while discharging real patients. A dedicated lecture during PGY 1 orientation was subsequently developed to highlight discharge needs to patients, and during morbidity and mortality conferences, known discharge errors are highlighted after incorporation of this training.

Our educational simulation was limited due to its small group of participants and the short time frame of deployment. The simulation was initially conducted before the SARS-CoV-2 pandemic. While it was anticipated to be a longitudinal curriculum targeting different groups of postgraduate learners, its scenarios were forced to transition to posthospital follow-up telemedicine encounters and subsequently had different learning objectives than the ones currently described. Should a follow-up iteration be conducted, incorporating the discharge practice early in training but performing the actual simulation encounter within 3–6 months of residency training could be beneficial, and repeating it close to the end of residency training, to truly link learner performance and self-assessment, would be advised. A telemedicine appointment scenario might also be beneficial for learners to assess patients postdischarge in self-reflection on the discharge process. Another limitation to this curriculum was the lack of incorporation of the social work discipline, due to the limited social work resources within our hospital system and academic center. Ideally, involving social work as an integral part of the development team, as well as the implementation team for feedback, would improve the implementation process and be another key component for learner feedback.

Continuing to conduct this simulation project can help us further understand the knowledge gaps that need to be closed to ensure safe discharges, especially for training physicians. This simulation has been adapted for and utilized with our transitional year program residents, though the postsimulation survey completion rate remains low due to its voluntary nature. The discharge checklist resonated with our learners and was subsequently developed into a reference tool within our EMR for all members of the health care teams when performing a hospital discharge. The emphasis on discharge practices also changed the format of our respective morbidity and mortality conferences, where residents could highlight difficulties or errors in hospital discharges. This simulation can be replicated with different learners, such as resident physicians from different specialties, or started earlier in undergraduate medical curricula or other health care professions. As pedagogic strategies evolve with new generations of learners, simulation-based learning is rapidly becoming a vital component of medical education. Active simulation-based training can equip residents with the skills necessary to discharge and transition patients safely and effectively into a postacute care setting.

## Appendices


Discharge Checklist Lecture.pptxPrebrief.docxSimulation Case 1.docxSimulation Case 2.docxSimulation Case Rubrics.docx
*All appendices are peer reviewed as integral parts of the Original Publication.*
