# Supplementary material for: Training to Transition: Using Simulation-Based Training to Improve Resident Physician Confidence in Hospital Discharges
Source: MedEdPORTAL. 2023 Sep 15;19:11348. doi: 10.15766/mep_2374-8265.11348 (PMC10502193; doi:10.15766/mep_2374-8265.11348)
Supplement: Supplementary file 1 — Discharge Checklist Lecture.pptxPrebrief.docxSimulation Case 1.docxSimulation Case 2.docxSimulation Case Rubrics.docx [file mep_2374-8265.11348-s001.zip › C. Simulation Case 1.docx]

**Appendix C: SP Case 1**

Date: July 1, 2019

Primary Case Author: Jenna N Sizemore, Spoorthi Sankineni, Andrea Bailey

Secondary Case Author: Maria Kolar, Shanthi Manivannan, Karen Clark, Sarah Sofka

Simulated Participant Educator: Jenna Sizemore

Name of Case: Case 1 – Hospital discharge to rural, home location

Name of educational and or assessment activity: Training to Transition: Using Simulation-based Training to Improve Resident Physician Confidence in Hospital Discharges

Challenge objective: Successfully discharge a patient with complex medical needs to a rural area

Domains:

⛝ Communication and Interpersonal skills

⛝ Medical History

⛝ Physical exam

⛝ Shared Decision Making

⛝ Patient Education

⛝ Clinical Reasoning

Type and level of learners: Post-graduate year 1 residents were utilized in our scenario, however, this case can be adapted for all level of clinician including medical students, advanced practice providers, physician assistant students, and modified for nursing students.

Case Objectives:

By the end of this activity, learners will be able to:

1. Identify relevant details from a discharge summary comprised of a brief patient history, hospital course, and medication list.

2. Enter patient discharge orders in an electronic medical record using a playground computer system.

3. Communicate accurate discharge orders about medications, outpatient appointments, and outpatient laboratory investigations to a patient and caregiver based on a discharge rubric with case-specific goals.

4. Respond effectively to patient and caregiver inquiries and concerns in a simulated discharge interaction

| SETTING: | Inpatient, Hospital room |
| --- | --- |
| PATIENT PROFILE: | |
| Age range | Adult patient, age need not be specific |
| Religious/spiritual background | Per simulated participant discretion |
| Sex (e.g., male, female, intersex, transwoman, transman) | Gender not specific to this case, all gender identities may be used |
| Sexual Orientation (e.g., heterosexual, lesbian, gay, bisexual, pansexual, queer, asexual) | Not specific to this case, all may be used. |
| Gender expression (e.g., man, woman, gender queer) | Not specified |
| Race/ethnicity: | All may be used |
| Physical description (e.g., BMI, height range) | Normal BMI, appropriate hygiene |
| Physical limitations | None |
| Patient appearance (e.g., disheveled, hospital gown, business casual, casual) | Hospital gown |
| Moulage + location (e.g., none, bruises, scars, body piercing, tattoos) | None |
| Affect (e.g., pleasant, cooperative) | Cooperative |
| Family group (e.g., who is family, who they live with) | Family member present at bedside, however, per SP discretion. Facilitators may considering developing a scenario with few family resources. Both scenarios depict a patient that lives alone. |
| Education | High school education |
| Level of health literacy | Minimal; simulated participant should prompt students to use layman’s terms if they start using medical jargon or abbreviations |
| Employment, if any - present and past, noting any current stresses | Currently works at the local gas station |
| Home/homeless - type of dwelling, number of stories, owned or rented | Rents a ranch-style house; steps present within |
| Financial situation- any current stresses | Does have financial stressors |
| Insurance Status (e.g., un/under/insured, public/private, HMO/PPO) | Insurance with a state-based insurance provider |
| Habits (i.e., diet, exercise, caffeine, smoking, alcohol, drugs) | Prior smoking history of 30 pack years, with smoking cessation 1 year prior to hospitalization.  Habitual caffeine consumption  No alcohol use  Minimal exercise and reduced exercise tolerance |
| Activities (i.e., hobbies, sports, clubs, friends) | None. |
| Typical day - what is the usual daily routine | Family member provides transportation to appointments when needed, but the patient manages own healthcare needs and requirements. The patient can drive but has difficulty accessing a vehicle and there is minimal public transportation available in their geographic location. Denies any food insecurity; prepares all meals and feels capable of continuing to do so. The patient does not have a close grocery store to her house and gets several items from a local gas station and does a large grocery store trip about once a month. Likes to read and watch television on for usual daily activities. |

| CASE INFORMATION | |
| --- | --- |
| Chief Concern: Hospital discharge after suffering a NSTEMI, with new diagnosis of coronary artery disease and ketosis prone diabetes mellitus type II. | Simulated Participant Questions:  “I’m worried about how I’ll do once I’m out of the hospital. Where do I go if I have any problems?” |
| Additional Concerns: Other, if any, concerns the patient has today (i.e., symptoms, requests, expectations, etc.) that will become part of set agenda. | “I am worried I won’t be able to make to all my follow up appointments.”  “Will someone be able to prescribe me all of the medicines I need when I leave the hospital?”  “Do I still need to take insulin? How do I give myself insulin?”    “Can we go over the diet I need eat once I’m back home?”  “Is it ok for me to shower where they did my heart procedure in my leg?”    **Lay caregiver:** (family member questions that can be asked)  “Does [relation to family member] have to go home with the IV in [their] arm?”  “Does [Jo] have activity restrictions?”    “What type of diet should [Jo] follow?”  “How often should [Jo] be checking her blood sugars [glucose]?”  “Can you explain exactly what happened to [Jo] while in the hospital?”  “[Jo] lives over 4 hours away, could you locate a clinic closer to home?” |
| THE PATIENT STORY: The SP will be asked to tell their symptom story and the personal and emotion impact for each of their concerns. You will want to write this is the patient voice. The symptom story should be able to answer this question: “Tell me more about [chief concern/additional concern], starting at the beginning and bringing me up to now.”    The personal context should be able to answer questions concerning the broader personal/psychosocial context of symptoms, especially the patient beliefs/attributions.    The emotional context should be able to ask how are you doing with this, how does this make you feel, how has this affected you emotionally? IMPACT: How has this affected your life? How has this been for your family? | I am an adult patient who is currently in the process of getting discharged from the hospital after being here for over 2 weeks. I have lived alone in a rural area for several years, and I work at a local gas station. My coworkers are great people who look out for me, as well as my family. I never needed to go to the doctor, as it was always so expensive, so I did not see a regular doctor for years before I was hospitalized.  About two weeks ago, I started getting having odd sensations of pressure in my chest and felt like I was close to passing out. I started peeing all the time and was always thirsty even though I was drinking a gallon of water a day. I passed out at work, and thankfully my coworker called an ambulance where they took me to our emergency department. The ER team found out my blood sugar was dangerously high and that I had a heart attack, and told me that I had to get transferred to another hospital. I was really nervous to be so far away from family. I am very grateful to the ER team because I feel like they saved my life.  I still feel weak and have lost weight while I’ve been recovering. I was told I am a diabetic now and that makes me feel uncomfortable because my mom had bad diabetes and had to have an amputation. I am worried I will have an amputation in the future. I’m nervous about my blood sugars going low, and I really hate needles and having to poke myself for insulin. I’ve been glad the nurses do it for me while I’m in the hospital, and they’ve really helped teach me how to do the injections myself.  I never had a regular doctor that I could go to before I came to the hospital, and now I’m worried about talking all these medications now.  I want to get back home as soon as I can but I’m scared that I might have another heart attack.  Lay caregiver role:  The lay caregiver is a family member who is very concerned about the health of their family member, and is interested in helping the family member return to home safely. They do not have a medical background, and may feel overwhelmed with worry at the seriousness of their family member’s illness. |
| HISTORY OF PRESENT ILLNESS/HOSPITAL COURSE: (The lay caregiver can answer some of these questions as well.) | |
| Adult patient admitted to a large academic hospital with an acute non ST elevation myocardial infarction and diabetic ketoacidosis. She received a left heart catheterization, with access through the right groin, with deployment of 2 drug eluting stents to her LAD and RCA. The patient was started on insulin and fluids for glucose control which resolved the diabetic ketoacidosis. The patient has continued on insulin injections, both basal and pre-prandial, through the hospitalization though it did take several days to achieve adequate control of blood glucose. The patient was started on dual antiplatelet therapy with Ticagrelor and Aspirin, as well as atorvastatin, metoprolol tartrate, and Lisinopril. Overall, the patient feels much improved. The left heart catheterization was tolerated well, without the development of any pseudoanuerysm or groin hematoma. | |
| Onset (when; gradual or sudden) | NA |
| Setting (what was going on or where was patient when symptoms first noticed?) | NA |
| Duration (how long) | Has been hospitalized for an extended period of time (> 2 weeks) |
| Time relationships (frequency, constant or intermittent) | NA |
| Location | NA |
| Radiation | NA |
| Quality | NA |
| Amount | NA |
| Aggravated by what | NA |
| Relieved by what | NA |
| Associated with what | NA |
| Attitude (what does the patient think is the problem, and how does he/she feel about it) | Nervous about discharge |
| Overall course | NA |
| REVIEW OF SYSTEMS: Significant positives and negatives | |
| General: Your clothes are fitting much looser so you feel that you’ve lost weight. No fever, chills or sweats. Appetite remains off though better than when you were first admitted.  **Eyes:** no vision changes.  **ENT:** No hearing changes  **Cardiovascular:** Your heart seems to beat faster and you’ve been a little winded with some of your normal activities since surgery.  **GU:** good bladder control  **MSK**: No joint pain or swelling.  **Dermatologic**: Very easy bruising.  **Psychiatric:** You are nervous and had been feeling depressed with your new medical diagnosis, but you are feeling slightly better now that you have recovered. You feel slight anxiety at the prospect of returning home. You do not have any auditory or visual hallucinations.  **Neurologic**: No numbness or tingling. No headaches. You feel overall weak and feel deconditioned since you’ve had limited activity during your hospitalization. | |
|  | |
| Past medical history | |
| Medication allergies (Name and reaction) | Penicillin (hives) |
| Environmental allergies (Name and reaction) | None |
| Illnesses | None prior to admission  Newly diagnosed during hospitalization  Coronary Artery Disease  Diabetes Type II, Ketosis prone  Hypertension  Hyperlipidemia |
| Vaccinations | Tdap updated  Covid updated |
| Surgeries | · Recent Left Heart Cath. |
| Accidents/ injuries/ trauma | · No major/traumatic injuries or trauma |
| Hospitalization | · Myocardial Infarction, DKA this admission |
|  | |
| Inclusive sexual and reproductive history | |
| Sexual practices  Sexual partners  Protection: Use of safer sex practices  Use of birth control if appropriate  Risk of intimate partner violence | Not applicable to case  Possible suggested history could include:  1 Prior pregnancy, term, complicated by gestation diabetes and pre-eclampsia. Not currently sexually active. |
| Ob/GYN HISTORY | Overall non-necessary to case:  A possible suggested history can include:  Age of onset of menses: 13, LMP 2 weeks ago  Age of menopause: NA  Number of pregnancies: 1  Number of live births: 1  Number of miscarriages: 0  Number of abortions 0 |
| Medications | - Glargine sub q injection, 45 units qhs (nightly) - Aspart sub q (subcutaneous) injection, 5 units pre-meal (before meals) - Aspirin 81 mg qd (daily) - Ticagrelor 90 mg bid (twice daily) - Lisinopril 2.5 mg qd (daily) - Metoprolol tartrate 25 mg bid (twice daily) - Atorvastatin 80 mg qd (daily) |
| Immunizations | Tdap – upon hospitalization  Covid – Pfizer, including all recommended boosters  Up to date  Unsure about additional vaccinations |
| Tobacco products: | Previous smoker – roughly 30 pack years |
| Alcohol | Never |
| Drugs | No lifetime use of opioids, marijuana, cocaine, methamphetamine use, or hallucinogens. Prior use of benzodiazepines taken from sister during a traumatic period of her life but none within three years. |
| Diet (describe) | The patient reports eating 2-3 times per day. Breakfast consists of cereal. Lunch is usually a sandwich or a salad. Sometimes the patient eats chips or cookies if they become hungry during the afternoon. Dinner usually has some meat, usually red meat or chicken. Frequent fast food use. Most of her meals are provided at her place of work. |
| Exercise (describe) | Able to exercise though feels deconditioned after hospitalization. |
| List any other important social history or information important to this case | The patient can drive but has limited access to a vehicle (shared among family members) and limited public transportation in their geographic location. |
| Family history |  |
| Mother, Father, Siblings, Grandparents, and other significant findings. | Father died of a heart attack in his 60’s  Mother died at 50 from a heart attack and had complications from diabetes.  The patient has one child who is reported to be healthy.  Siblings are reported to be healthy. |
| Physical Exam    The learners may ask the SP to stand up, walk, turn around and sit down to check balance and ambulation. SPs should perform this task well.    If available, the SP should have a device depicting a peripheral intravenous catheter. When asked, the patient or lay caregiver can ask if this can be removed before discharge. The catheter entry site looks healthy, without discharge or any erythema around the site.  The patient will be in a hospital gown and will have a normal exam. A bandage can be placed in the groin if needed to suggest a prior access site from the recent left heart catheterization.    The SP should be certain to mention these things in layman’s terms as potential clues if prompted.  -Facial features: dark circles under eyes  -Mouth: May have some concerns about cavities  -Musculoskeletal: No joint pain, can move all joints without difficulty  -Extremities: Inquires if the IV line can be removed  -Skin: pale, may also show concern about several bruises, or discuss easy bruising | |
| PHYSICAL EXAM FINDINGS |  |
| 1) Written in layman’s terms | The patient is sitting comfortably. |
| 2) General appearance- affect, appearance, position of patient at opening (i.e. sitting, laying down, holding abdomen etc.) | Nervous affect, appropriate hygiene. Sitting on hospital bed. |
| 3) Vital signs | Normal (e.g. a learner can take a blood pressure, pulse, and temperature) |
| 4) Specific findings and affect | a. Psychiatric- Slightly nervous  b. An IV line may still be in place with the goal of the learner discussing with the patient removal before discharge |
| 5) Response to certain physical movements | No limitations to activity. |
| DIAGNOSIS AND DIFFERENTIAL |  |
| Diagnosis with support from positive and negative history and PE findings | Coronary artery disease  Diabetes mellitus type II –Ketosis prone |
| Differential with support from positive and negative history and PE findings | Differential N/A |
| MANAGEMENT OR DIAGNOSTIC PLAN | Prescribe all medications on hospital discharge  Recommend to remove PICC line or IV catheter before discharge  Discuss follow up with a primary care provider and/or a specialist (Cardiology, Endocrinology) that is within distance for the patient to receive care  Discuss hospital appointments needed after discharge  Discuss red flags/warning signs related to patient’s medications or medical problems  Discuss the hospitalization and transitional plan of care with the lay caregiver  Place orders for discharge without medication errors |
| PROFESSIONALISM ISSUES OR CHALLENGES: | During the simulation, it can be challenging for learners to budget their time appropriately, and the lay caregiver can ask questions to move the case forwarded if needed. |
