# Supplementary material for: Training to Transition: Using Simulation-Based Training to Improve Resident Physician Confidence in Hospital Discharges
Source: MedEdPORTAL. 2023 Sep 15;19:11348. doi: 10.15766/mep_2374-8265.11348 (PMC10502193; doi:10.15766/mep_2374-8265.11348)
Supplement: Supplementary file 1 — Discharge Checklist Lecture.pptxPrebrief.docxSimulation Case 1.docxSimulation Case 2.docxSimulation Case Rubrics.docx [file mep_2374-8265.11348-s001.zip › D. Simulation Case 2.docx]

**Appendix D: SP Case 2**

Date: July 1, 2019

Primary Case Author: Jenna N Sizemore, Spoorthi Sankineni, Andrea Bailey

Secondary Case Author: Maria Kolar, Shanthi Manivannan, Karen Clark, Sarah Sofka

Simulated Participant Educator: Jenna Sizemore

Name of Case: Case 2 – Hospital discharge to rural, home location

Name of educational and or assessment activity: Training to Transition: Using Simulation-based Training to Improve Resident Physician Confidence in Hospital Discharges

Challenge objective: Successfully discharge a patient with complex medical needs to a rural area

Domains:

⛝ Communication and Interpersonal skills

⛝ Medical History

⛝ Physical exam

⛝ Shared Decision Making

⛝ Patient Education

⛝ Clinical Reasoning

Type and level of learners: Post-graduate year 1 residents were utilized in our scenario, however, this case can be adapted for all level of clinician including medical students, advanced practice providers, physician assistant students, and nursing students.

Case Objectives:

By the end of this activity, learners will be able to:

1. Identify relevant details from a discharge summary comprised of a brief patient history, hospital course, and medication list.

2. Enter patient discharge orders in an electronic medical record using a playground computer system.

3. Communicate accurate discharge orders about medications, outpatient appointments, and outpatient laboratory investigations to a patient and caregiver based on a discharge rubric with case-specific goals.

4. Respond effectively to patient and caregiver inquiries and concerns in a simulated discharge interaction

| SETTING: | Inpatient, Hospital room |
| --- | --- |
| PATIENT PROFILE: | |
| Age range | Adult patient, age need not be specific |
| Religious/spiritual background | Per simulated participant discretion |
| Gender (e.g., male, female, intersex, transwoman, transman) | Gender not specific to this case, all gender identities may be used. |
| Sexual Orientation (e.g., heterosexual, lesbian, gay, bisexual, pansexual, queer, asexual) | Not specific to this case, all may be used. |
| Gender expression (e.g., man, woman, gender queer) | Not specified |
| Race/ethnicity: | All may be used |
| Physical description (e.g., BMI, height range) | Normal BMI, appropriate hygiene |
| Physical limitations | None |
| Patient appearance (e.g., disheveled, hospital gown, business casual, casual) | Hospital gown |
| Moulage + location (e.g., none, bruises, scars, body piercing, tattoos) | None |
| Affect (e.g., pleasant, cooperative) | Cooperative |
| Family group (e.g., who is family, who they live with) | Family member present at bedside, however, per SP discretion. Facilitators may considering developing a scenario with few family resources. Both scenarios depict a patient that lives alone. |
| Education | High school education |
| Level of health literacy | Minimal; simulated participant should prompt students to use layman’s terms if they start using medical jargon or abbreviations |
| Employment, if any - present and past, noting any current stresses | Currently unemployed, previously worked retail |
| Home/homeless - type of dwelling, number of stories, owned or rented | Rents a ranch-style house; steps present within |
| Financial situation- any current stresses | Does have financial stressors |
| Insurance Status (e.g., un/under/insured, public/private, HMO/PPO) | Insurance with a state-based insurance provider |
| Habits (i.e., diet, exercise, caffeine, smoking, alcohol, drugs) | Prior smoking history of 3 pack years, without current tobacco exposure. Prior opioid use, including injection use, now in early remission. Prior use of benzodiazepines but no recent use within the past 3 years. No history of alcohol use. |
| Activities (i.e., hobbies, sports, clubs, friends) | None. |
| Typical day - what is the usual daily routine | Family member provides transportation to appointments when needed, but the patient manages own healthcare needs and requirements. The patient currently no longer has an active driver’s license but does use public transportation if available. Denies any food insecurity; prepares all meals and feels capable of continuing to do so. The patient does not have a close grocery store to their house and gets several items from a local gas station; they usually perform a large grocery store trip about once a month. Likes to read and watch television on for usual daily activities. |

| CASE INFORMATION | |
| --- | --- |
| Chief Concern: Hospital discharge after an extensive (> 6 week) hospitalization for *Methicillin resistant staphylococcus aureus* bacteremia. | Simulated Participant Questions:  “I’m worried about how I’ll do once I’m out of the hospital. Where do I go if I have any problems?” |
| Additional Concerns: Other, if any, concerns the patient has today (i.e., symptoms, requests, expectations, etc.) that will become part of set agenda. | “I am worried I won’t be able to make to all my follow up appointments.”  “Will someone be able to prescribe me all of the medicines I need when I leave the hospital?”    “Can we go over the diet I need eat once I’m back home?”    “I can’t drive, what do you I do if I can’t get to the lab to get my blood drawn?”    “Is there someone I can call if I need any help once I’m back home?”    **Lay caregiver:** (family member questions that can be asked)  “Does [Jo] have to go home with the IV in [their] arm?”  “Does [Jo] have activity restrictions?”    “What type of diet should [Jo] follow?”  “What foods does [Jo] need to avoid while taking Warfarin?”  “I thought the anticoagulation educator told me [Jo] would need to have frequent blood work to monitor PT/INR, how often does this need done, where should [they] have their blood drawn. Who should we contact for those results?”  “[Jo] lives over 4 hours away, could you locate a clinic closer to home?” |
| THE PATIENT STORY: The SP will be asked to tell their symptom story and the personal and emotion impact for each of their concerns. You will want to write this is the patient voice. The symptom story should be able to answer this question: “Tell me more about [chief concern/additional concern], starting at the beginning and bringing me up to now.”    The personal context should be able to answer questions concerning the broader personal/psychosocial context of symptoms, especially the patient beliefs/attributions.    The emotional context should be able to ask how are you doing with this, how does this make you feel, how has this affected you emotionally? IMPACT: How has this affected your life? How has this been for your family? | I am an adult patient who is currently in the process of getting discharged from the hospital after being here for over 6 weeks. I have had difficulties avoiding pain pills and heroin since I was in my teenage years, but I was always able to keep up with my job and family until recently. I’ve wanted to stop using heroin for a long time but I’ve had to keep using it to stop the withdrawal, which was so bad I could not function.  About two months ago, I started getting fevers and chills and felt so weak I couldn’t walk, which is why I went to the emergency department close to home. The ER team found an infection in my blood stream. I had to come up here to get surgery to get the infection off my heart valve which was really scary for me. My family has been supportive of me and helps me make all my appointments. I’m happy I’ve been able to get started on treatment. I’m really worried I’ll relapse if I don’t get my medication when I leave the hospital.  I still feel weak and have lost weight while my body was healing from this infection, but I feel more like myself than I have in a long time. The therapy I was getting in the hospital really helped me.  I never had a regular doctor that I could go to before I came to the hospital, and now I’m worried that I won’t have my therapist when I’m back home helping me.  I want to start finding a job as soon as I get home because I really like having a schedule to keep me centered on my recovery.  Lay caregiver role:  The lay caregiver is a family member who is very concerned about the health of their family member, and is interested in helping the family member return to home safely. They do not have a medical background, and may feel overwhelmed with worry at the seriousness of their family member’s illness. |
| HISTORY OF PRESENT ILLNESS/HOSPITAL COURSE: (The lay caregiver can answer some of these questions as well.) | |
| Adult patient admitted to a large academic hospital with *Methicillin-resistant staph aureus* with recent exposure to injected opioids was received as a transfer from a rural, critical access hospital several weeks ago. The patient was ultimately diagnosed with aortic valve endocarditis with embolic phenomenon, including CNS (central nervous system - embolic brain and spinal) lesions. They received a successful mechanical valve replacement. The patient completed 6 weeks of therapy with IV Vancomycin, started on buprenorphine-naloxone, and they are currently in clinical remission for opioid use disorder. The patient received cognitive behavioral therapy while admitted and completed 6 weeks of antibiotics for the infection. Anticoagulation was initiated with warfarin for the mechanical aortic valve. The patient received pharmacist-led education about different foods that may interact with warfarin. The patient is very excited to be closer to family after spending six weeks in the hospital. The patient lives alone in a rural county. The patient is currently unemployed but is hopeful to find work once closer to home. The patient uses a state based insurer for their medical insurance. A family member is with the patient on the day of discharge. | |
| Onset (when; gradual or sudden) | NA |
| Setting (what was going on or where was patient when symptoms first noticed?) | NA |
| Duration (how long) | Has been hospitalized for an extended period of time (> 4-6 weeks) |
| Time relationships (frequency, constant or intermittent) | NA |
| Location | NA |
| Radiation | NA |
| Quality | NA |
| Amount | NA |
| Aggravated by what | NA |
| Relieved by what | NA |
| Associated with what | NA |
| Attitude (what does the patient think is the problem, and how does he/she feel about it) | Nervous about discharge |
| Overall course | NA |
| REVIEW OF SYSTEMS: Significant positives and negatives | |
| General: Your clothes are fitting much looser so you feel that you’ve lost weight. No fever, chills or sweats. Appetite remains off though better than when you were first admitted.  **Eyes:** no vision changes.  **ENT:** No hearing changes  **Cardiovascular:** Your heart seems to beat faster, and you’ve been a little winded with some of your normal activities since surgery. Sometimes your incision on your chest aches.  **GU:** good bladder control  **MSK**: No joint pain or swelling.  **Dermatologic**: Very easy bruising.  **Psychiatric:** You are nervous and had been feeling depressed but you are beginning to feel better, and you think your medications and therapy have really helped with your depression. No current thoughts of hurting yourself.  **Neurologic**: No numbness or tingling. No headaches. You feel overall weak from feeling deconditioned from not being as active while hospitalized. | |
|  | |
| Past medical history | |
| Medication allergies (Name and reaction) | Penicillin (hives) |
| Environmental allergies (Name and reaction) | None |
| Illnesses | Major depression  Generalized Anxiety |
| Vaccinations | Tdap updated  Covid updated  Patient is unsure of other vaccination status. |
| Surgeries | · Open heart surgery, aortic valve replacement |
| Accidents/ injuries/ trauma | · No major/traumatic injuries or trauma |
| Hospitalization | · MRSA bacteremia with aortic valve endocarditis |
|  | |
| Inclusive sexual and reproductive history | |
| Sexual practices  Sexual partners  Protection: Use of safer sex practices  Use of birth control if appropriate  Risk of intimate partner violence | Not applicable  No current or prior pregnancies |
| Ob/GYN HISTORY | Overall, not applicable to the current scenario.  A possible history can include:  Age of onset of menses: 13, LMP 2 weeks ago  Age of menopause: NA  Number of pregnancies: 0  Number of live births: 0  Number of miscarriages: 0  Number of abortions 0 |
| Medications | ● Warfarin 2.5 mg daily Mon, Wed, Fri, 3 mg daily Tues, Thur, Sat  ● Sertraline 100 mg daily  ● Buprenorphine-Naloxone 8 mg/2 mg daily  ● Buspirone 30 mg daily |
| Immunizations | Tdap – upon hospitalization  Covid – Pfizer, including all recommended boosters  Up to date |
| Tobacco products: | Previous smoker – roughly 3 pack years |
| Alcohol | Never |
| Drugs | Recent severe use of opioids, including heroin and prescription opioids, with both inhalational and injection use.  No marijuana, cocaine, methamphetamine use. Prior use of benzodiazepines but none within three years. |
| Diet (describe) | The patient reports eating 2-3 times per day. Breakfast consists of cereal. Lunch is usually a sandwich or a salad. Sometimes the patient eats saltines or butter cookies if hungry during the afternoon. Dinner usually has some meat, usually red meat or chicken. Frequent fast food use. Limited access to a grocery store and usually obtains all food once monthly in a large grocery trip, with mostly preserved foods. |
| Exercise (describe) | Able to exercise though feels deconditioned after hospitalization. |
| List any other important social history or information important to this case | The patient is no longer driving as they do not currently have a license. They have some, though limited, public transportation available in their geographic location. A family member can assist with transportation if needed on most occasions. |
| Family history |  |
| Mother, Father, Siblings, Grandparents, and other significant findings. | Father died of a heart attack in his 60’s  Mother died at 40 from a car accident. PMH of Depression.  The patient does not have any children.  Siblings are reported to be healthy. |
| Physical Exam    The learners may ask the SP to stand up, walk, turn around and sit down to check balance and ambulation. SPs should perform this task well.    If available, the SP should have a device depicting of peripherally inserted central venous catheter or a peripheral intravenous catheter. When asked, the patient or lay caregiver can ask if this can be removed before discharge. There will be no discharge or erythema concerning for infection around the site.  The patient will be in a hospital gown and will have a normal exam, though the learner may mention the absence of a heart murmur or cardiac click, as it may be expected to have this finding after open heart surgery with a valve replacement.    The SP should be certain to mention these things in layman’s terms as potential clues if prompted.  -Facial features: dark circles under eyes  -Mouth: May have some concerns about cavities  -Musculoskeletal: No joint pain, can move all joints without difficulty  -Extremities: Inquires if the IV line can be removed  -Skin: pale, may also show concern about several bruises, or discuss easy bruising if asked by the learner | |
| PHYSICAL EXAM FINDINGS |  |
| 1) Written in layman’s terms | The patient is sitting comfortably. |
| 2) General appearance- affect, appearance, position of patient at opening (i.e. sitting, laying down, holding abdomen etc.) | Nervous affect, appropriate hygiene. Sitting on hospital bed. |
| 3) Vital signs | Normal (e.g. a student will take blood pressure, pulse, and temperature) |
| 4) Specific findings and affect | a. Psychiatric- Slightly nervous  b. An IV line may still be in place with the goal of the learner discussing with the patient removal before discharge |
| 5) Response to certain physical movements | No limitations to activity. |
| DIAGNOSIS AND DIFFERENTIAL |  |
| Diagnosis with support from positive and negative history and PE findings | Aortic valve endocarditis with successful clearance of bacteremia, ready for discharge home. |
| Differential with support from positive and negative history and PE findings | Differential N/A |
| MANAGEMENT OR DIAGNOSITIC PLAN | Prescribe all medications on hospital discharge  Recommend to remove PICC line or IV catheter before discharge  Discuss follow up with a primary care provider and/or a specialist in medication-assisted treatment for opioid use disorder that is within distance for the patient to receive care. Additionally can discuss the transition for continuation of therapy services, or discuss the use of telemedicine to maintain the current therapy schedule.  Discuss hospital appointments needed after discharge  Discuss red flags/warning signs related to patient’s medications or medical problems  Discuss the hospitalization and transitional plan of care with the lay caregiver  Place orders for discharge without medication errors |
| PROFESSIONALISM ISSUES OR CHALLENGES: | During the simulation, it can be challenging for learners to budget their time appropriately and the lay caregiver can ask questions to move the case forwarded if needed. |
