# Supplementary material for: Training to Transition: Using Simulation-Based Training to Improve Resident Physician Confidence in Hospital Discharges
Source: MedEdPORTAL. 2023 Sep 15;19:11348. doi: 10.15766/mep_2374-8265.11348 (PMC10502193; doi:10.15766/mep_2374-8265.11348)
Supplement: Supplementary file 1 — Discharge Checklist Lecture.pptxPrebrief.docxSimulation Case 1.docxSimulation Case 2.docxSimulation Case Rubrics.docx [file mep_2374-8265.11348-s001.zip › E. Simulation Case Rubrics.docx]

| **Discharge Rubric**  **CASE 1**  (New diagnosis of DM II, CAD)  Author: Andrea Bailey, MSN, FNP-BC  Total Points: _____/42 | |
| --- | --- |
| **Reason for Hospitalization and Red Flags:** 8 points | |
| **Reason for Hospitalization:** Review of:   - new diagnoses - procedure(s) - labs - acute - brief review of chronic follow-up needs, etc. | Fully Addressed (5 points.):____    Semi-Addressed (3 points):____    Did not Address (0 point):_____    Overall Score: |
| **Red Flags** and **Hospital Point of Contact for Questions:**  Hypoglycemia:   - sweating - feeling hungry - passing out, etc.    Hyperglycemia:   - very thirsty - drinking a lot   **Contact**:   - Transition Team member or PCP, or DC service) | Fully Addressed (3 points.):____    Semi-Addressed (1 points):____    Did not Address (0 point):_____    Overall Score: |
| **Education and After Visit Summary (written) Instructions:** 14 points | |
| **Diet Instructions:**   - Diabetic Diet - Diabetes Educator | Fully Addressed (2 points.):____    Semi-Addressed (1 points):____    Did not Address (0 point):_____    Overall Score: |
| **Activity Instructions:**   - Activity as Tolerated | Fully Addressed (2 points.):____    Semi-Addressed (1 points):____    Did not Address (0 point):_____    Overall Score: |
| **Wounds Instructions:**     - PCI site-7 days old-no special instructions | Fully Addressed (2 points.):____    Semi-Addressed (1 points):____    Did not Address (0 point):_____    Overall Score: |
| **Medication Instructions:**   Insulin Specific:   - dose - side effects - red flags - who to call and when - when to seek emergent treatment; - Hypoglycemia counseling - blood glucose log - how to administer and check blood glucose | Fully Addressed (8 points.):____    Semi-Addressed (4 points):____    Did not Address (0 point):_____    Overall Score: |
| **Follow-Up Appointments and Post-Hospitalization Care:** 17 points | |
| **PCP:**   - Proximity to Appts.   (resides greater than 2 hours away)   - Transportation - PCP Obtained - Visit Scheduled Prior to Discharge - Describing discharge plan of care to patient and lay caregiver - Geographic Proximity to Follow up clinics | Fully Addressed (6 points.):____    Semi-Addressed (3 points):____    Did not Address (0 point):_____    Overall Score: |
| **Specialist(s):**   - Cardiology | Fully Addressed (2 points.):____    Semi-Addressed (1 points):____    Did not Address (0 point):_____    Overall Score: |
| **Lay Care Giver Point of Contact for Questions:** | Fully Addressed (2 pts.):_____    Semi-Addressed (1 points):____    Did not Address (0 point):_____    Overall Score: |
| **Wound/Medication Supplies:**   - Testing Strips - Lancets | Fully Addressed (2 points.):____    Semi-Addressed (1 points):____    Did not Address (0 point):_____    Overall Score: |
| **Durable Medical Equipment:**     - Glucometer - Diabetic testing supplies | Fully Addressed (2 points.):____    Semi-Addressed (1 points):____    Did not Address (0 point):_____    Overall Score: |
| **Unique Patient Needs:**   - lives alone - new medications - limited access to grocery store | Fully Addressed (3 points.):____    Semi-Addressed (1 points):____    Did not Address (0 point):_____    Overall Score: |
| **Medication Obtainment:** 3 points | |
| - Medication affordable? - Is a Prior-Authorization Needed? (potentially for Insulin/Ticagrelor; diabetic supplies - Can patient obtain medications at a local pharmacy in the future? | Fully Addressed (3 points.):____    Semi-Addressed (1 points):____    Did not Address (0 point):_____    Overall Score: |

| **Discharge Instruction Category:**  **Discharge SIM CASE 2**  (New diagnosis of OUD on MAT and new start anticoagulation)   Author: Andrea Bailey, MSN, FNP-BC    Total Points: _____/41 | |  |
| --- | --- | --- |
| **Reason for Hospitalization and Red Flags:** 6 points | |  |
| **Reason for Hospitalization:**   - Review of new diagnose(s) - Procedure(s) - Labs - brief review of follow-up needs | Fully Addressed (4 pts.):_____    Semi-Addressed (2 points):____    Did not Address (0 point):_____    Overall Score:  ______ |  |
| **Red Flags** and **Hospital Point of Contact for Questions:**     - Hospital DC Service - Transition Team member | Fully Addressed (2 pts.):_____    Semi-Addressed (1 points):____    Did not Address (0 point):_____    Overall Score: _____ |  |
| **Education and After Visit Summary (written) Instructions:** 22 points | |  |
| **Diet Instructions:**    Coumadin:   - Vitamin K intake-same amt. each day - limit alcohol - Medication interactions - Laboratory monitoring | Fully Addressed (4 pts.):_____    Semi-Addressed (2 points):____    Did not Address (0 point):_____    Overall Score:______ |  |
| **Activity Instructions:**     - Post-Operative Special Instructions - Currently advanced to full activity by surgical team | Fully Addressed (2 pts.):_____    Semi-Addressed (1 points):____    Did not Address (0 point):_____    Overall Score:   _________ |  |
| **Wounds Instructions:**     - **Old PICC site** - Discuss removal, counsel on warning signs of developing redness, swelling - **Sternal incision instructions** | Fully Addressed (4 pts.):_____    Semi-Addressed (2 points):____    Did not Address (0 point):_____    Overall Score:   ________ |  |
| **Medication Instructions:**    **Coumadin**:   - Special Instructions: (reason for prescription;  take exactly as prescribed;   Discuss with provider before taking any new meds  Current Dose   - Next Dose - Side Effect - When to seek emergent Treatment - Next Lab Draw Time and Place - Which provider is following the results? | Fully Addressed (8 pts.):_____    Semi-Addressed (4 points):____    Did not Address (0 point):_____    Overall Score:   _______ |  |
| **Unique Patient Needs:**     - Referral to continue CBT - behavioral medicine follow-up if not provided by PCP - community resources - Transportation limitations, discussion of telemedicine usage if needed. | Fully Addressed (4 pts.):_____    Semi-Addressed (2 points):____    Did not Address (0 point):_____    Overall Score: |  |
| **Follow-Up Appointments and Post-Hospitalization Care:** 12 points | |  |
| **PCP:**     - PCP Verified - Visit Scheduled Prior to Discharge - Transportation - Verify Hand-Off to PCP - Geographic Proximity to follow up clinic or current hospital | Fully Addressed (5 pts.):_____    Semi-Addressed (3 points):____    Did not Address (0 point):_____    Overall Score: |  |
| **Specialist(s):**     - Confirm appointments and patients ability to follow with - Cardiac Surgery - Behavioral Medicine/Psychology - Primary care provider | Fully Addressed (5 pts.):_____    Semi-Addressed (3 points):____    Did not Address (0 point):_____    Overall Score:_____ |  |
| **Lay Care Giver Point of Contact for Questions:**     - Discuss transitional plan of care with the lay caregiver - Answer questions from the laycaregiver | Fully Addressed (2 pts.):_____    Semi-Addressed (1 points):____    Did not Address (0 point):_____    Overall Score: ______ |  |
| **Medication Obtainment**: 3 points | |  |
| - Can Patient Afford? - Is a Prior-Authorization Needed? - Can providers that patient will follow with be able to continue prescribing current medication? | Fully Addressed (3 pts.):_____    Semi-Addressed (1 points):____    Did not Address (0 point):_____    Overall Score:_____  Comments: |  |
