# Supplementary material for: Training to Transition: Using Simulation-Based Training to Improve Resident Physician Confidence in Hospital Discharges
Source: MedEdPORTAL. 2023 Sep 15;19:11348. doi: 10.15766/mep_2374-8265.11348 (PMC10502193; doi:10.15766/mep_2374-8265.11348)
Supplement: Supplementary file 1 — Discharge Checklist Lecture.pptxPrebrief.docxSimulation Case 1.docxSimulation Case 2.docxSimulation Case Rubrics.docx [file mep_2374-8265.11348-s001.zip › A. Discharge Checklist Lecture.pptx]

## Slide 1
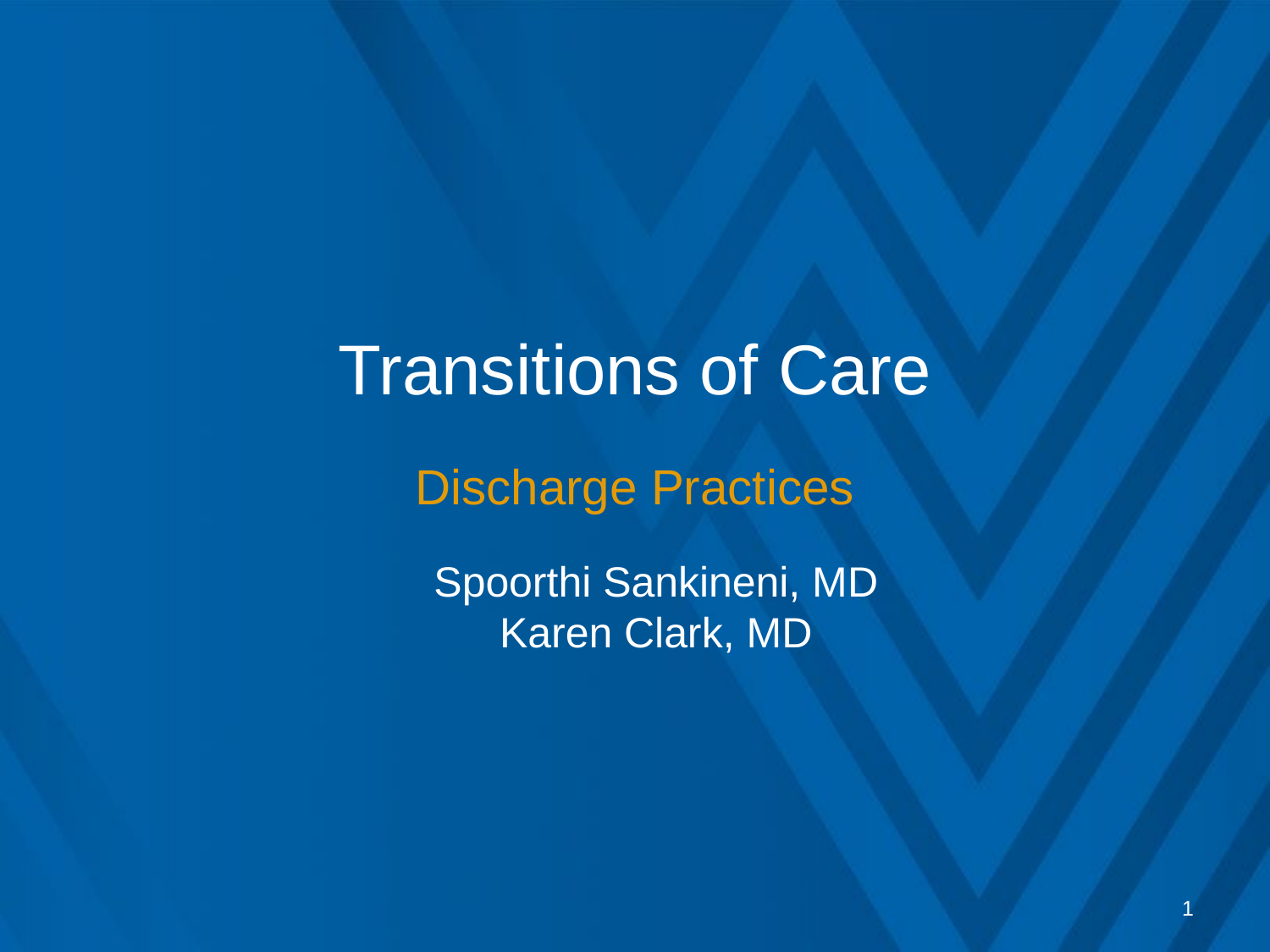

# Transitions of Care
Discharge Practices
Spoorthi Sankineni, MD
Karen Clark, MD
1

## Slide 2
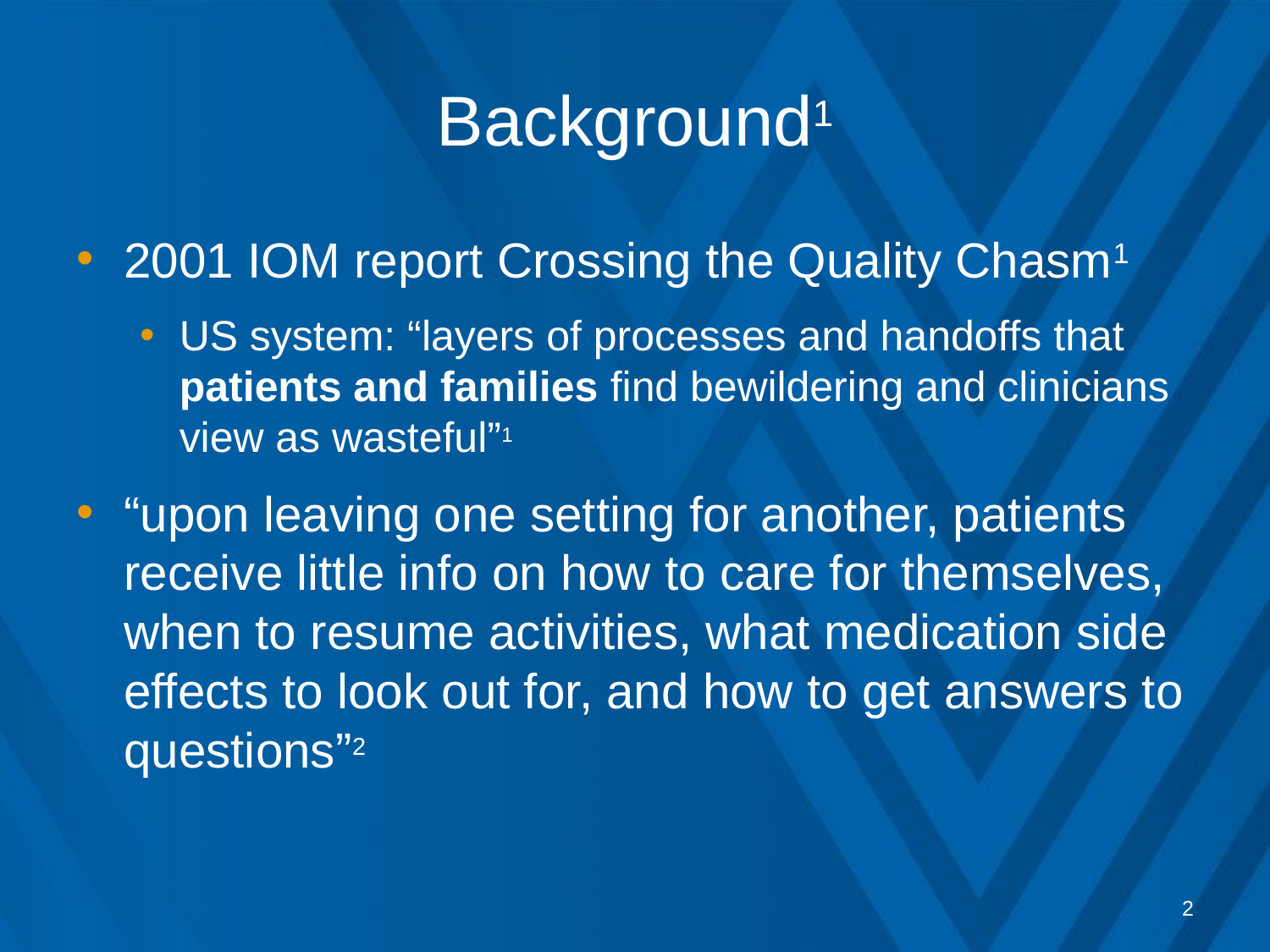

# Background1
2001 IOM report Crossing the Quality Chasm1
US system: “layers of processes and handoffs that patients and families find bewildering and clinicians view as wasteful”1
“upon leaving one setting for another, patients receive little info on how to care for themselves, when to resume activities, what medication side effects to look out for, and how to get answers to questions”2
2

## Slide 3
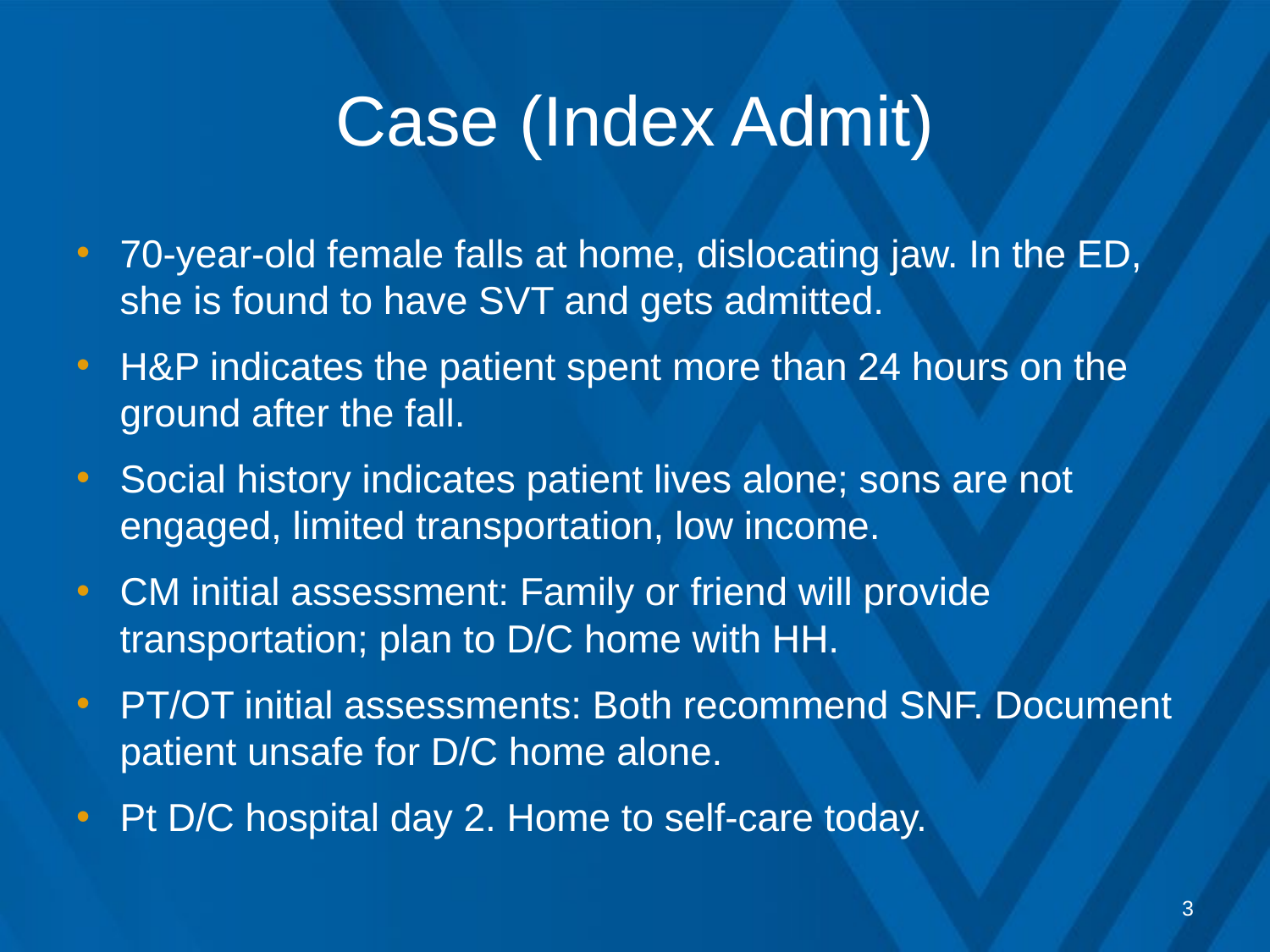

# Case (Index Admit)
70-year-old female falls at home, dislocating jaw. In the ED, she is found to have SVT and gets admitted.
H&P indicates the patient spent more than 24 hours on the ground after the fall.
Social history indicates patient lives alone; sons are not engaged, limited transportation, low income.
CM initial assessment: Family or friend will provide transportation; plan to D/C home with HH.
PT/OT initial assessments: Both recommend SNF. Document patient unsafe for D/C home alone.
Pt D/C hospital day 2. Home to self-care today.
3

## Slide 4
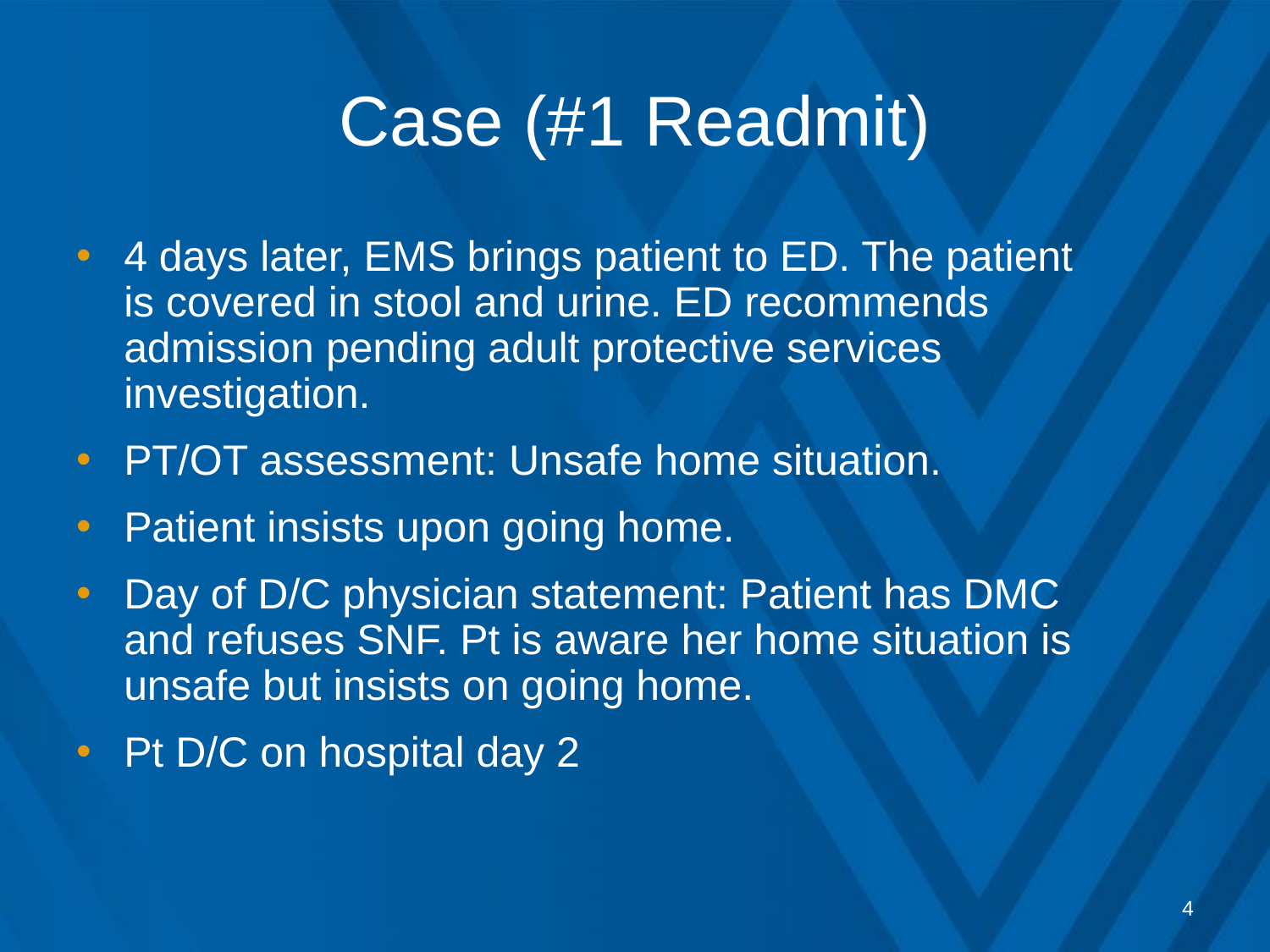

# Case (#1 Readmit)
4 days later, EMS brings patient to ED. The patient is covered in stool and urine. ED recommends admission pending adult protective services investigation.
PT/OT assessment: Unsafe home situation.
Patient insists upon going home.
Day of D/C physician statement: Patient has DMC and refuses SNF. Pt is aware her home situation is unsafe but insists on going home.
Pt D/C on hospital day 2
4

## Slide 5
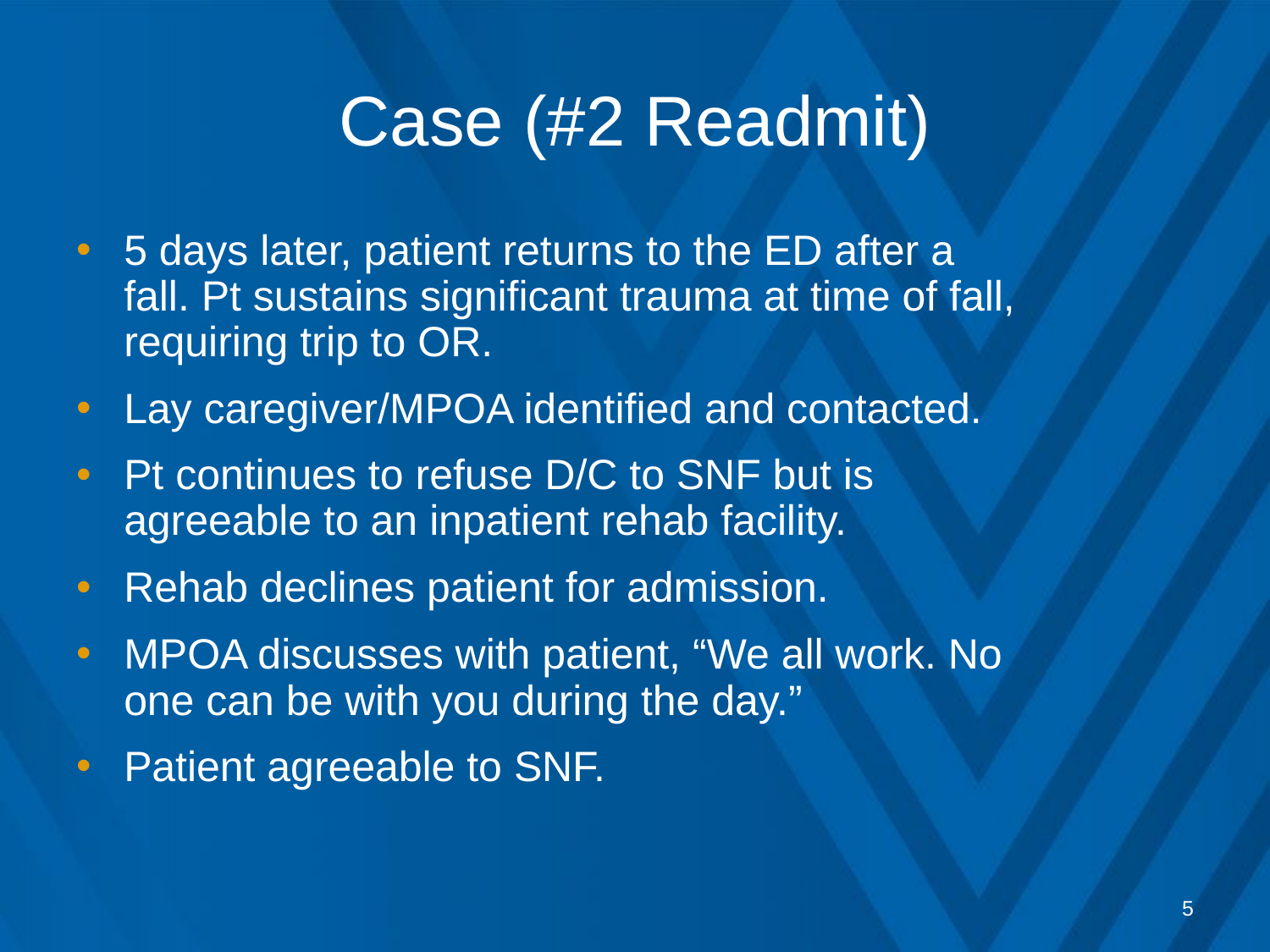

# Case (#2 Readmit)
5 days later, patient returns to the ED after a fall. Pt sustains significant trauma at time of fall, requiring trip to OR.
Lay caregiver/MPOA identified and contacted.
Pt continues to refuse D/C to SNF but is agreeable to an inpatient rehab facility.
Rehab declines patient for admission.
MPOA discusses with patient, “We all work. No one can be with you during the day.”
Patient agreeable to SNF.
5

## Slide 6
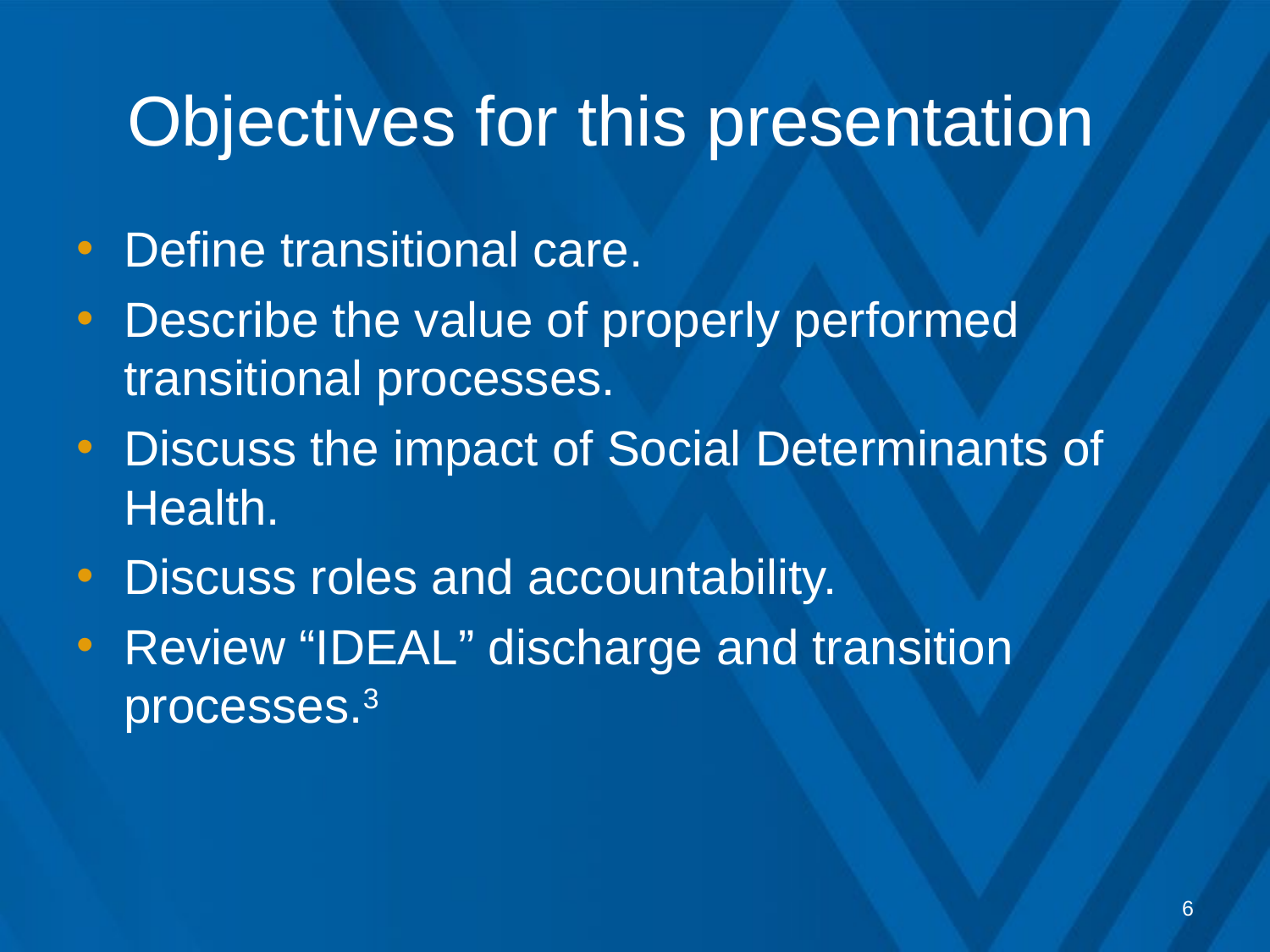

# Objectives for this presentation
Define transitional care.
Describe the value of properly performed transitional processes.
Discuss the impact of Social Determinants of Health.
Discuss roles and accountability.
Review “IDEAL” discharge and transition processes.3
6

## Slide 7
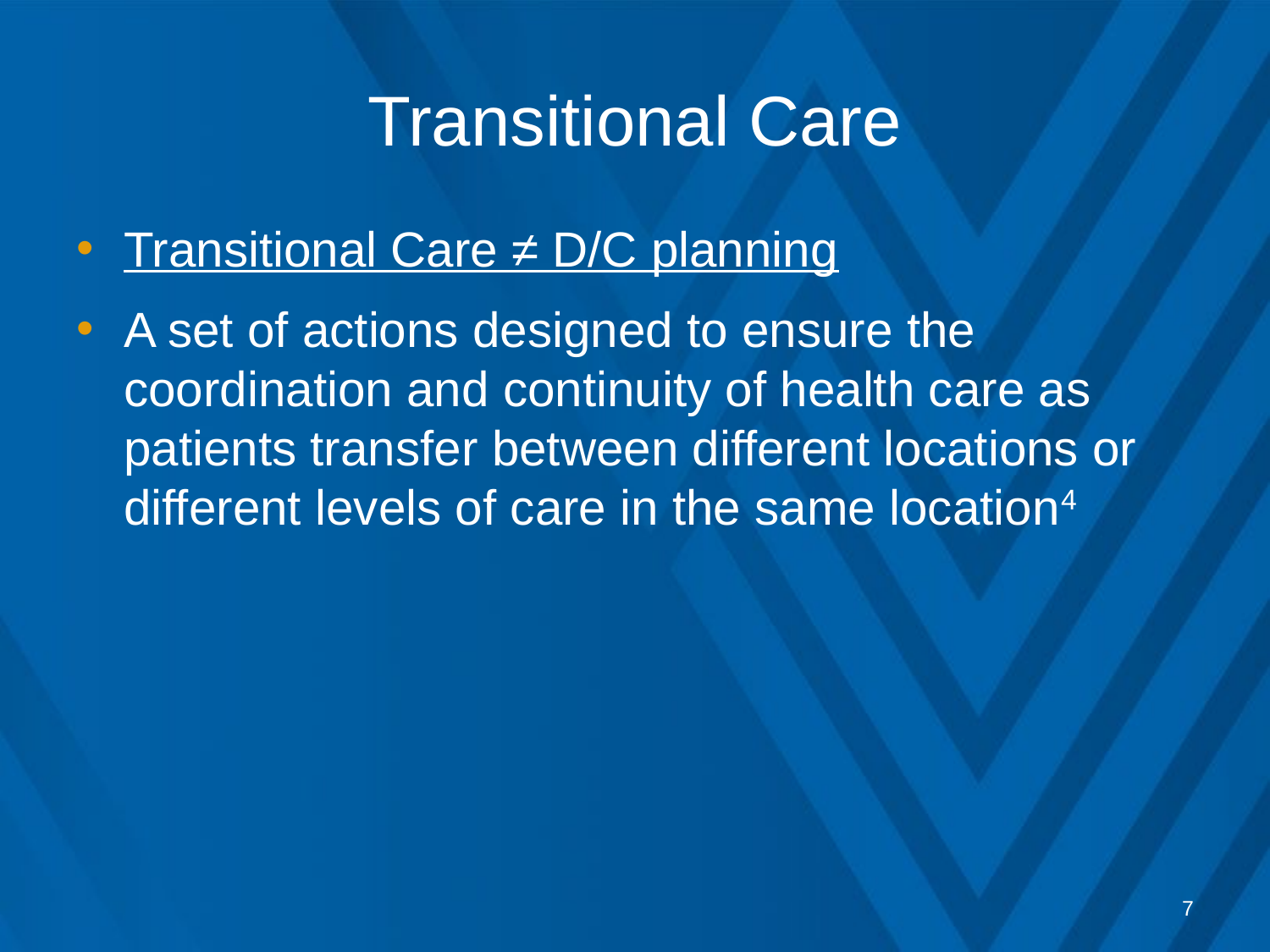

# Transitional Care
Transitional Care ≠ D/C planning
A set of actions designed to ensure the coordination and continuity of health care as patients transfer between different locations or different levels of care in the same location4
7

## Slide 8
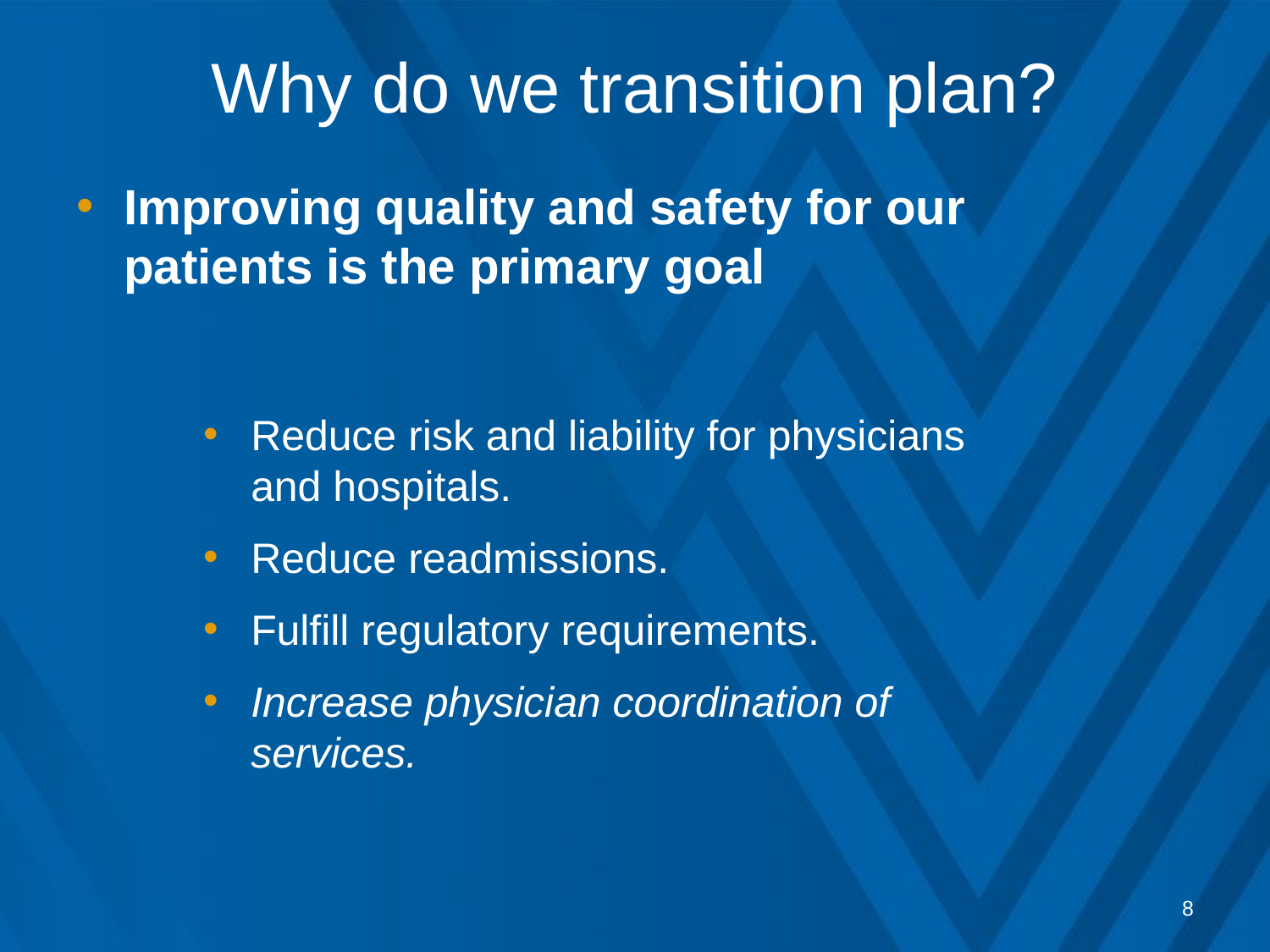

# Why do we transition plan?
Improving quality and safety for our patients is the primary goal
Reduce risk and liability for physicians and hospitals.
Reduce readmissions.
Fulfill regulatory requirements.
Increase physician coordination of services.
8

## Slide 9
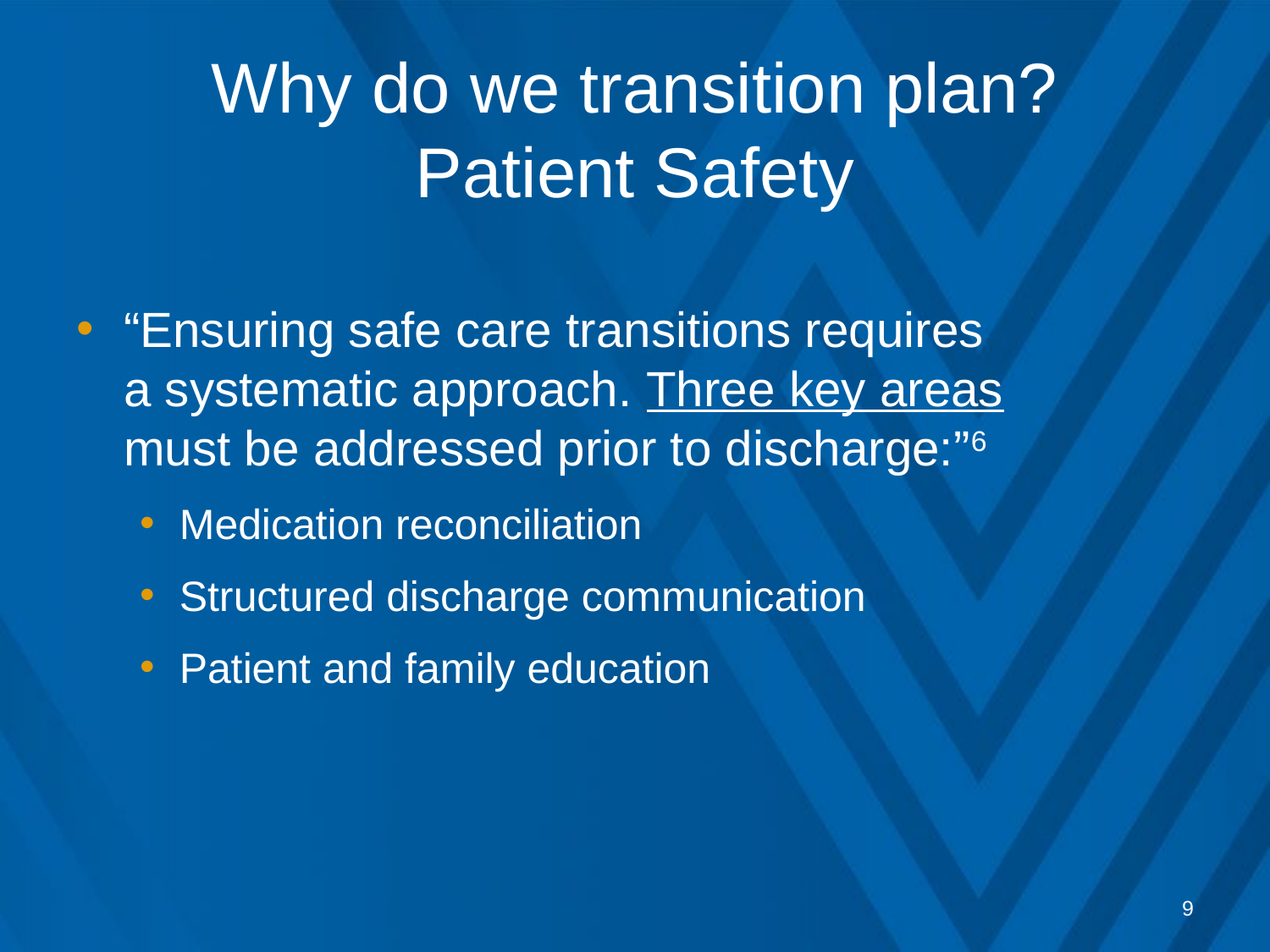

# Why do we transition plan? Patient Safety
“Ensuring safe care transitions requires a systematic approach. Three key areas must be addressed prior to discharge:”6
Medication reconciliation
Structured discharge communication
Patient and family education
9

## Slide 10
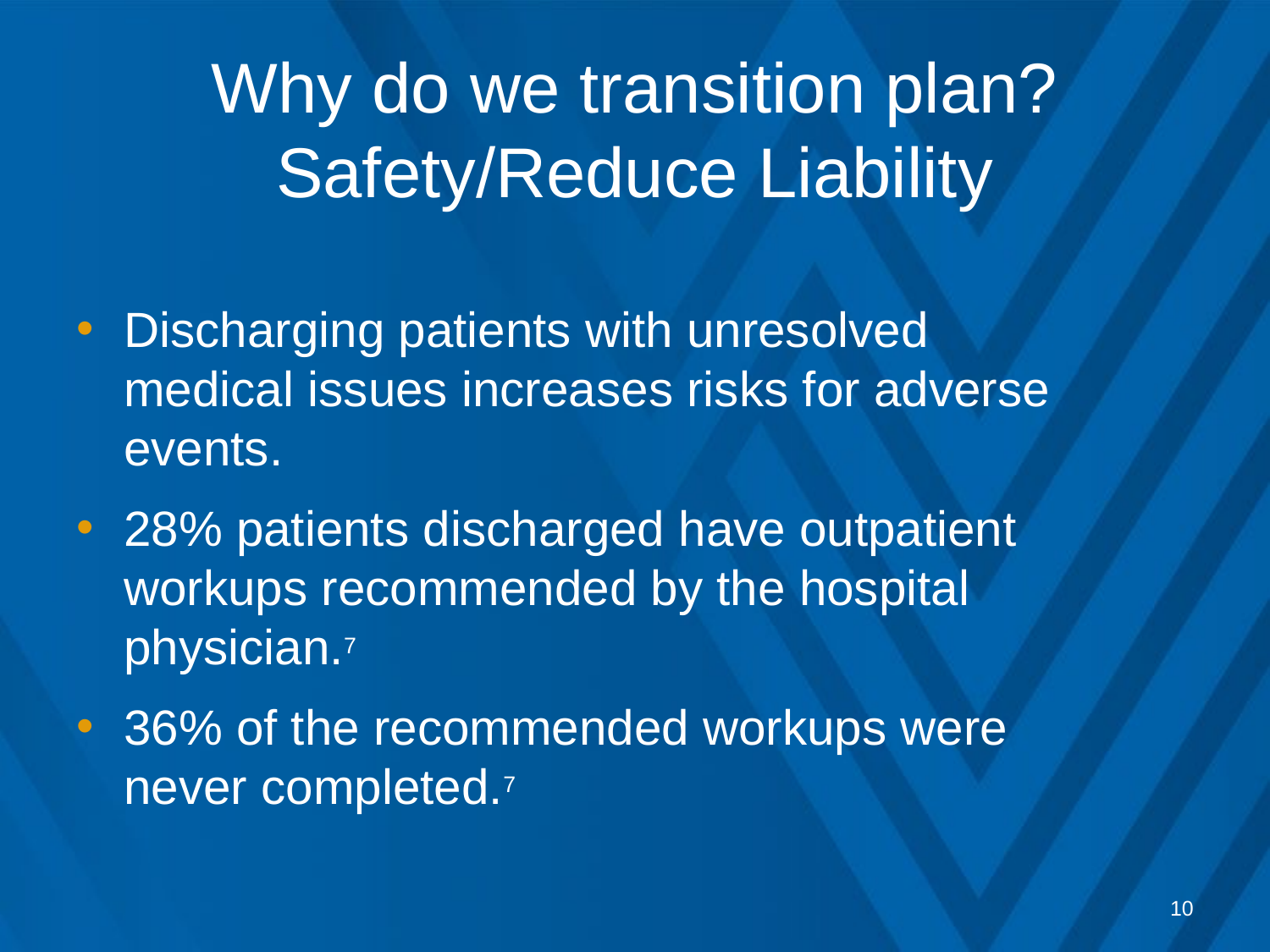

# Why do we transition plan? Safety/Reduce Liability
Discharging patients with unresolved medical issues increases risks for adverse events.
28% patients discharged have outpatient workups recommended by the hospital physician.7
36% of the recommended workups were never completed.7
10

## Slide 11
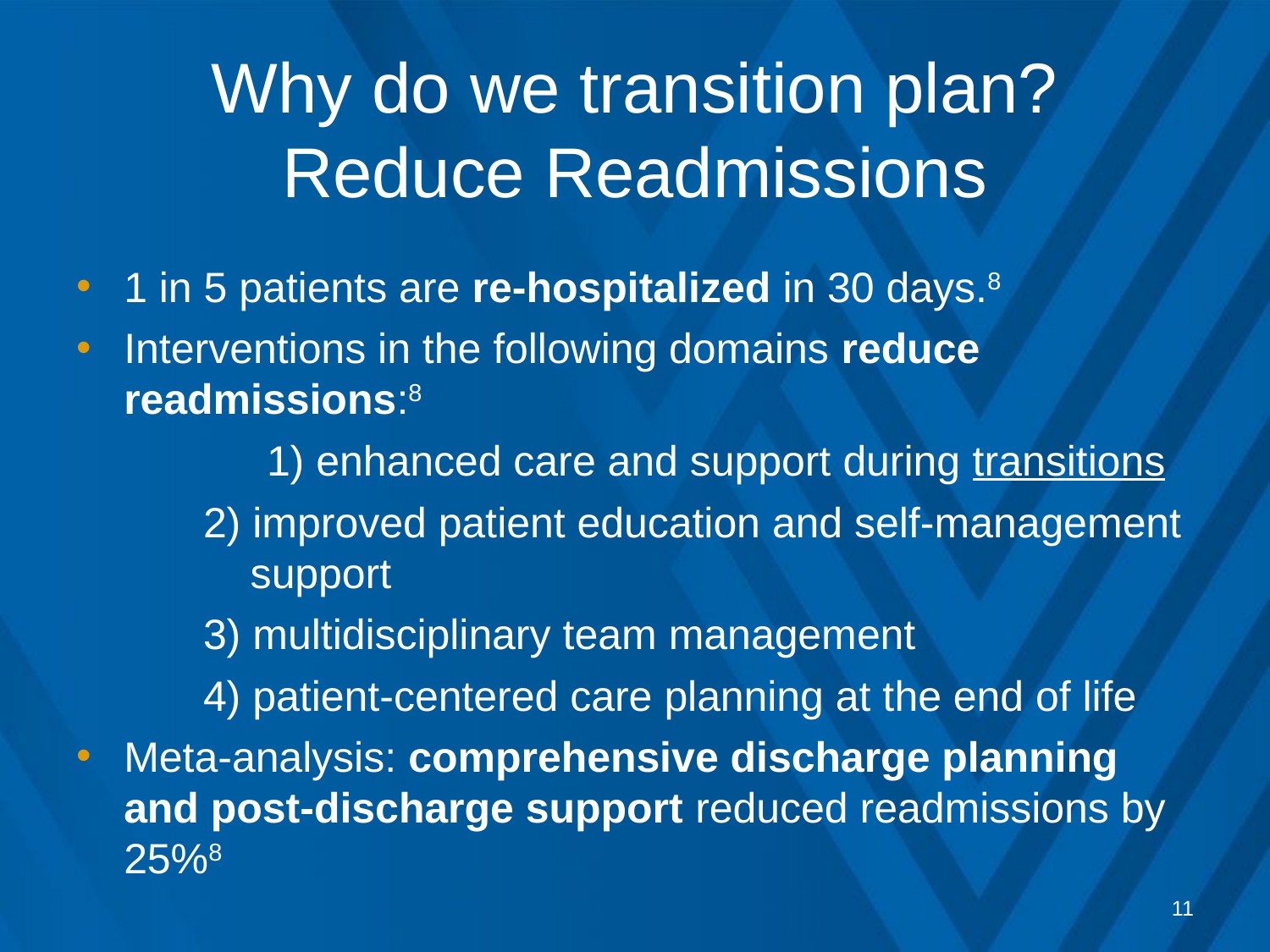

# Why do we transition plan? Reduce Readmissions
1 in 5 patients are re-hospitalized in 30 days.8
Interventions in the following domains reduce readmissions:8
	1) enhanced care and support during transitions
	2) improved patient education and self-management 	 support
	3) multidisciplinary team management
	4) patient-centered care planning at the end of life
Meta-analysis: comprehensive discharge planning and post-discharge support reduced readmissions by 25%8
11

## Slide 12
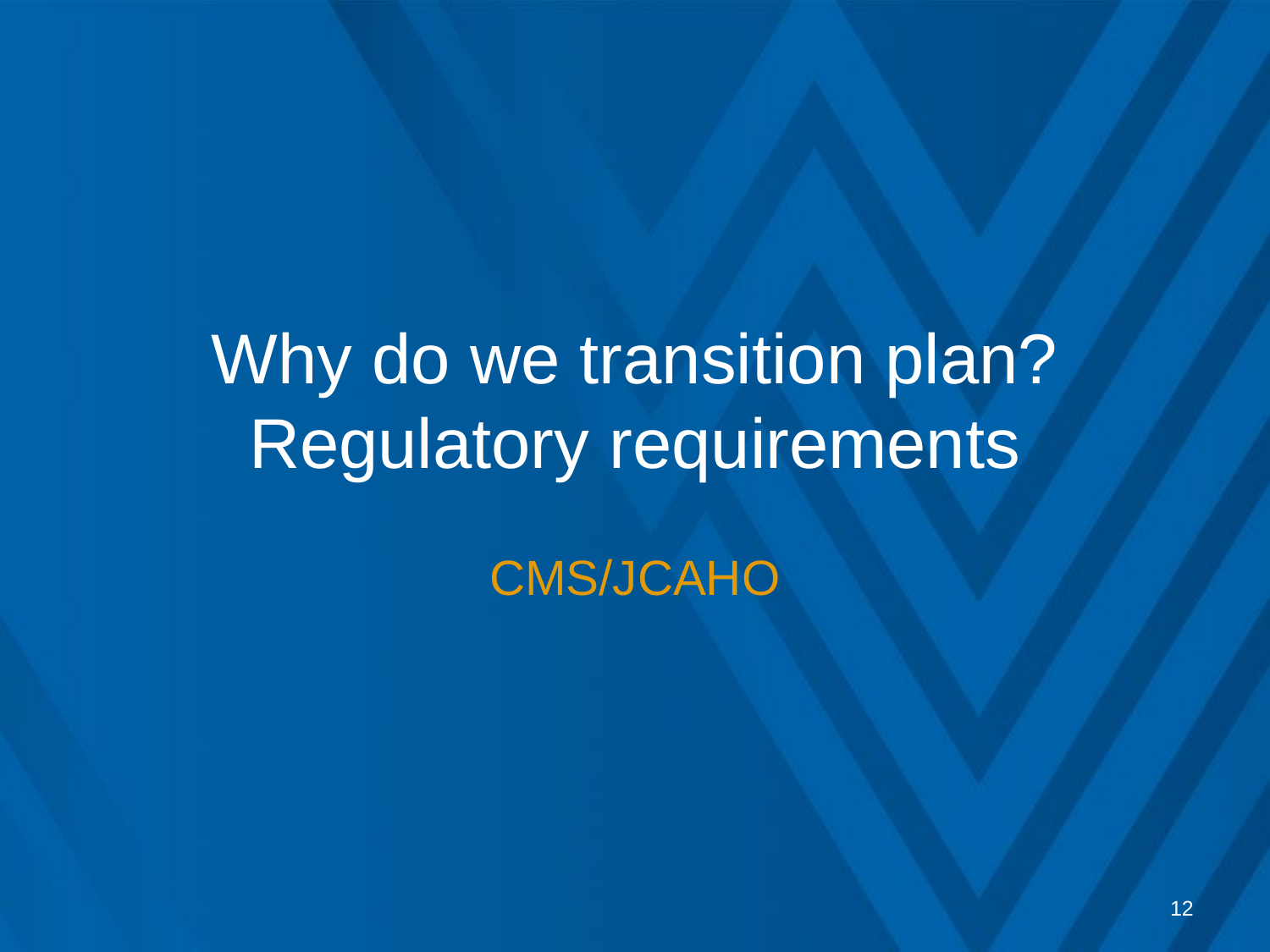

# Why do we transition plan? Regulatory requirements
CMS/JCAHO
12

## Slide 13
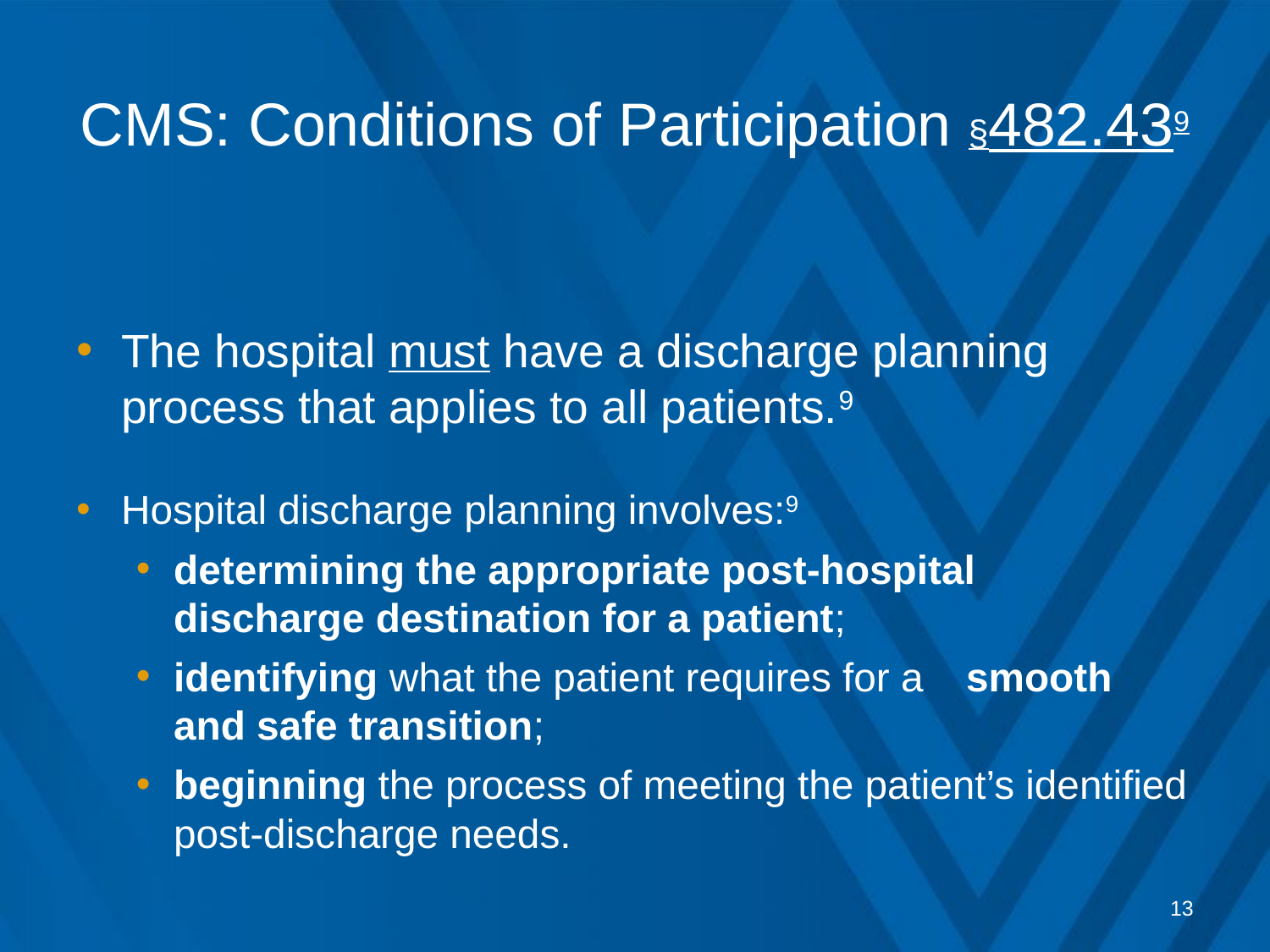

# CMS: Conditions of Participation §482.439
The hospital must have a discharge planning process that applies to all patients.9
Hospital discharge planning involves:9
determining the appropriate post-hospital 	discharge destination for a patient;
identifying what the patient requires for a 	smooth and safe transition;
beginning the process of meeting the patient’s identified post-discharge needs.
13

## Slide 14
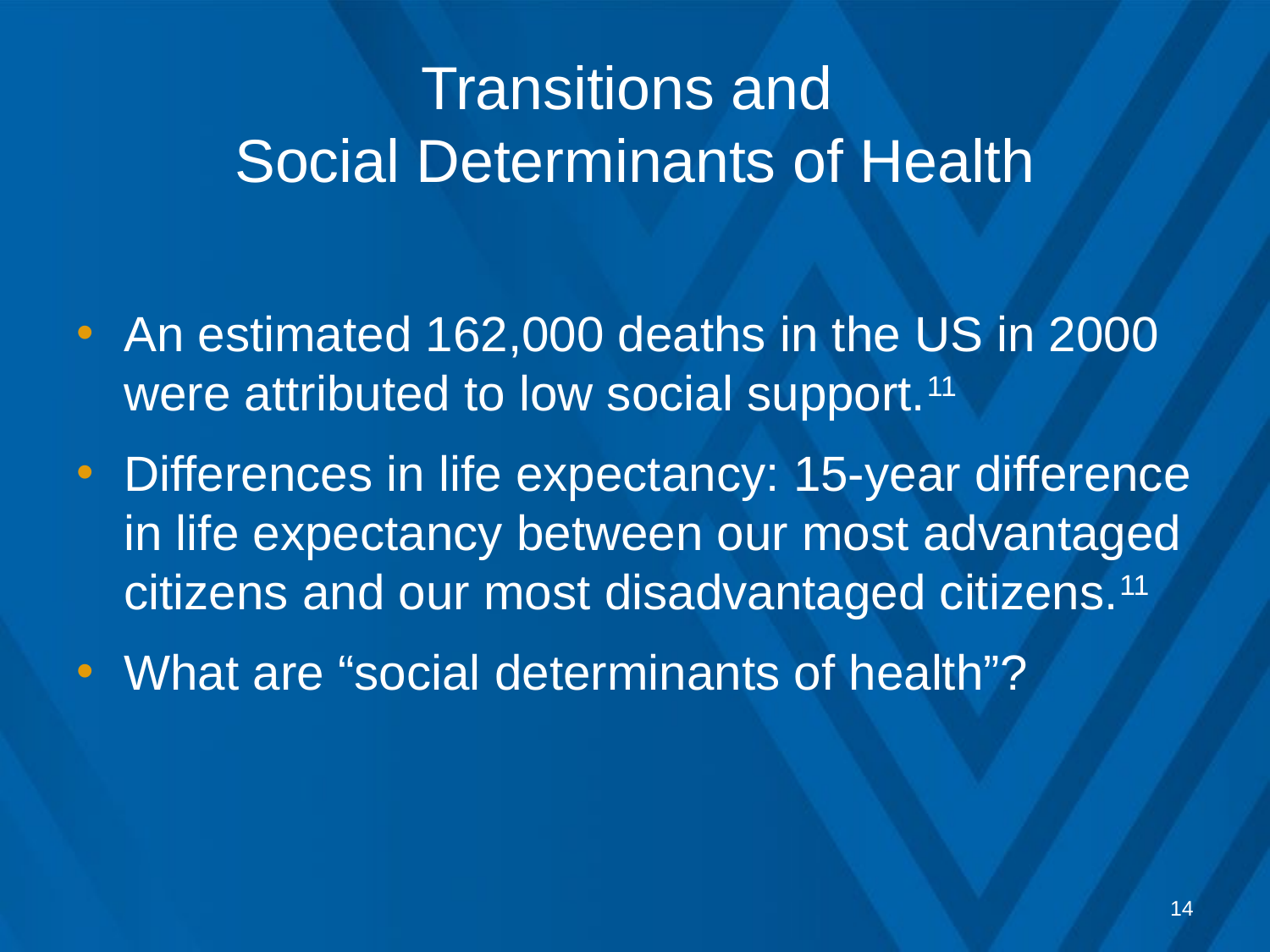

# Transitions and Social Determinants of Health
An estimated 162,000 deaths in the US in 2000 were attributed to low social support.11
Differences in life expectancy: 15-year difference in life expectancy between our most advantaged citizens and our most disadvantaged citizens.11
What are “social determinants of health”?
14

## Slide 15
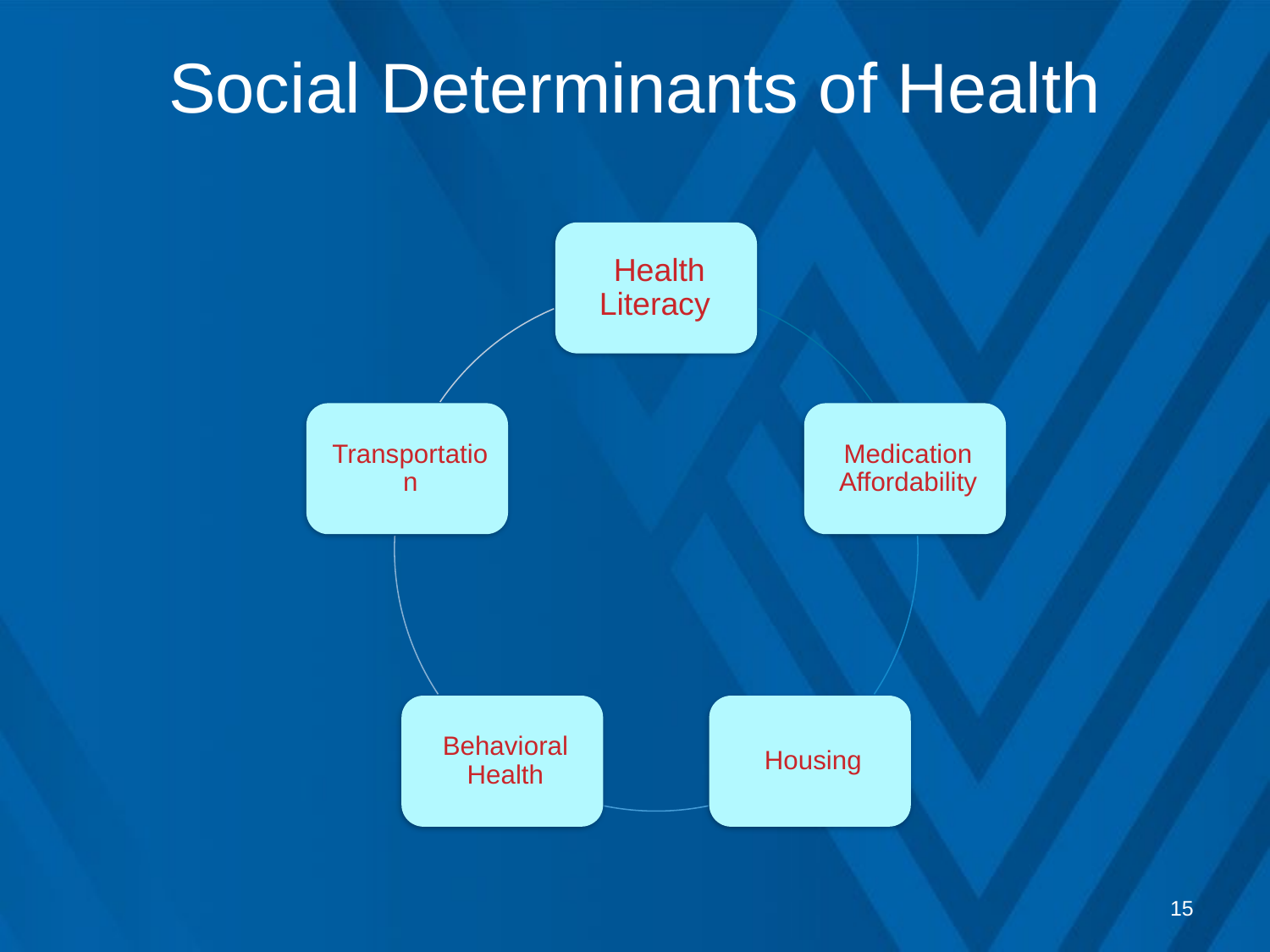

# Social Determinants of Health
15

## Slide 16
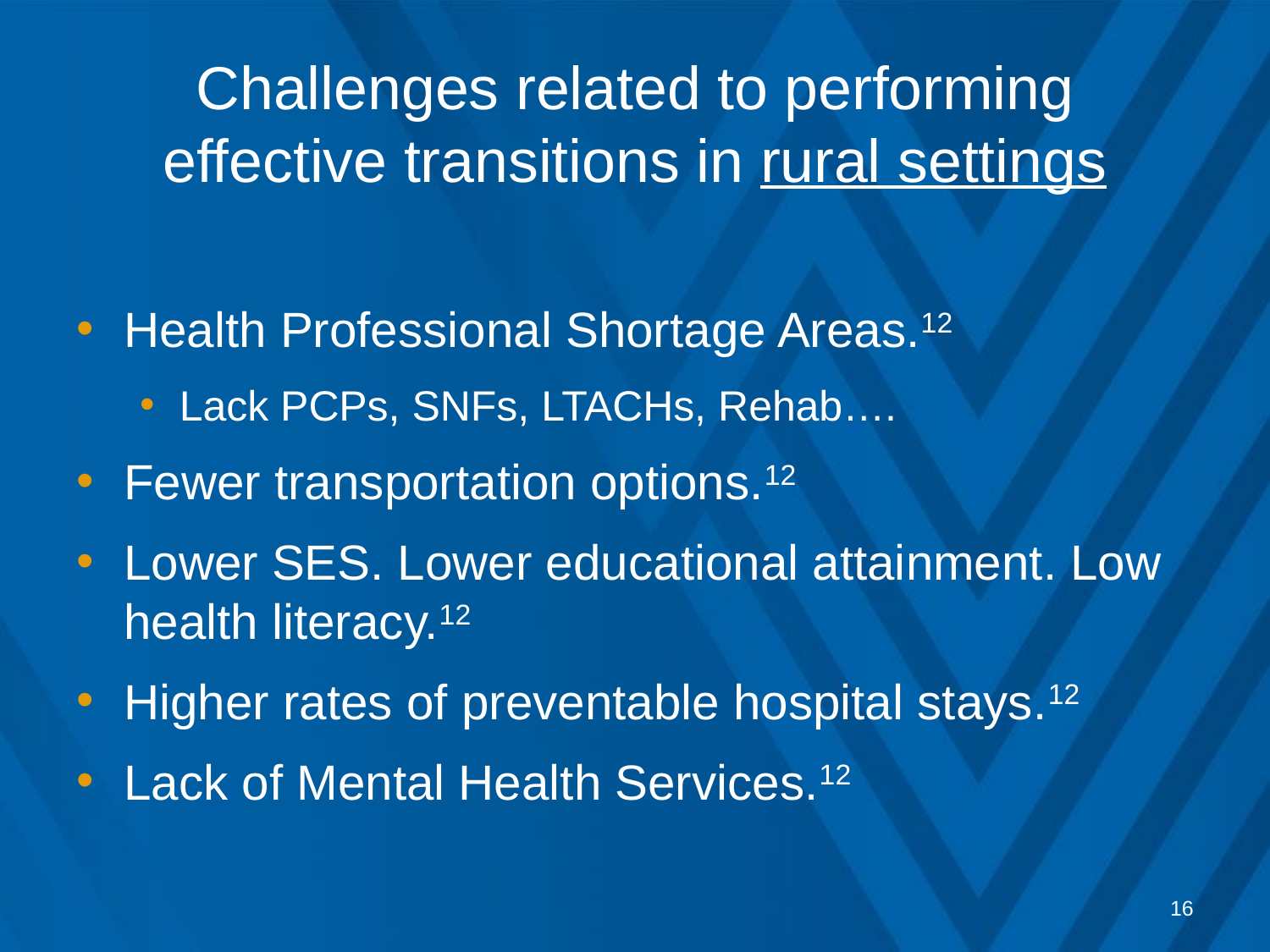

# Challenges related to performing effective transitions in rural settings
Health Professional Shortage Areas.12
Lack PCPs, SNFs, LTACHs, Rehab….
Fewer transportation options.12
Lower SES. Lower educational attainment. Low health literacy.12
Higher rates of preventable hospital stays.12
Lack of Mental Health Services.12
16

## Slide 17
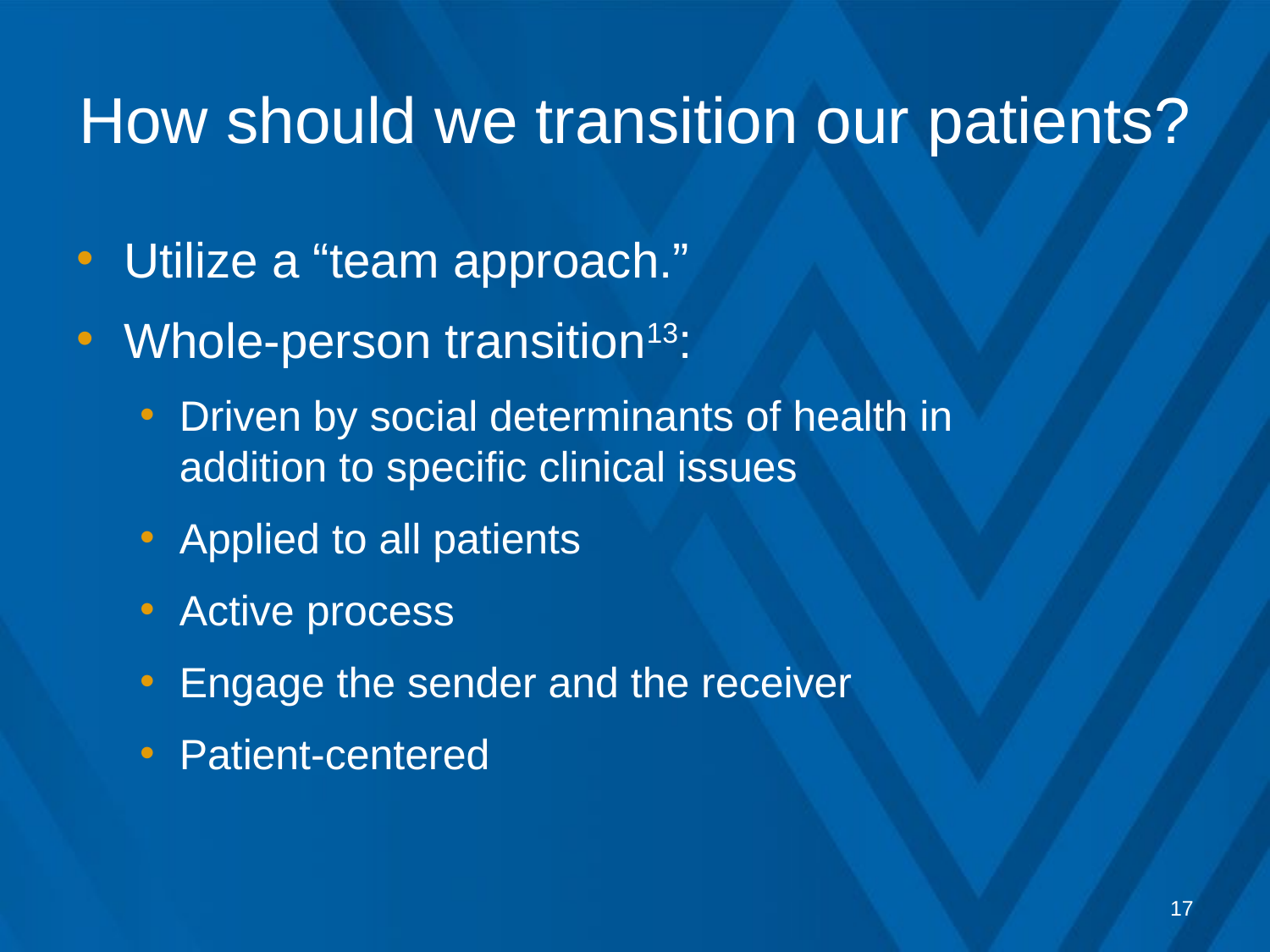

# How should we transition our patients?
Utilize a “team approach.”
Whole-person transition13:
Driven by social determinants of health in addition to specific clinical issues
Applied to all patients
Active process
Engage the sender and the receiver
Patient-centered
17

## Slide 18
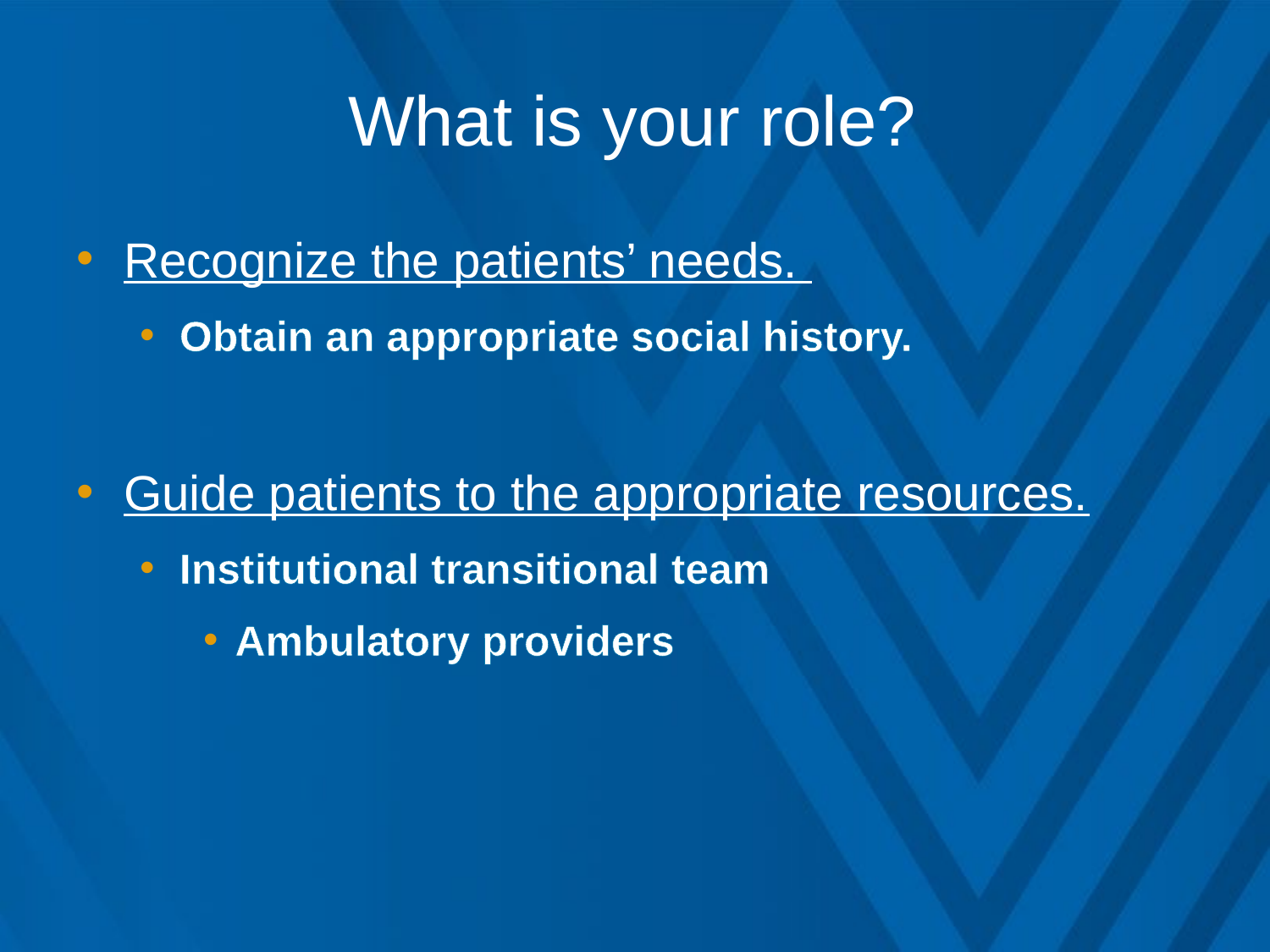

# What is your role? [1]
Recognize the patients’ needs.
Obtain an appropriate social history.
Guide patients to the appropriate resources.
Institutional transitional team
Ambulatory providers

## Slide 19
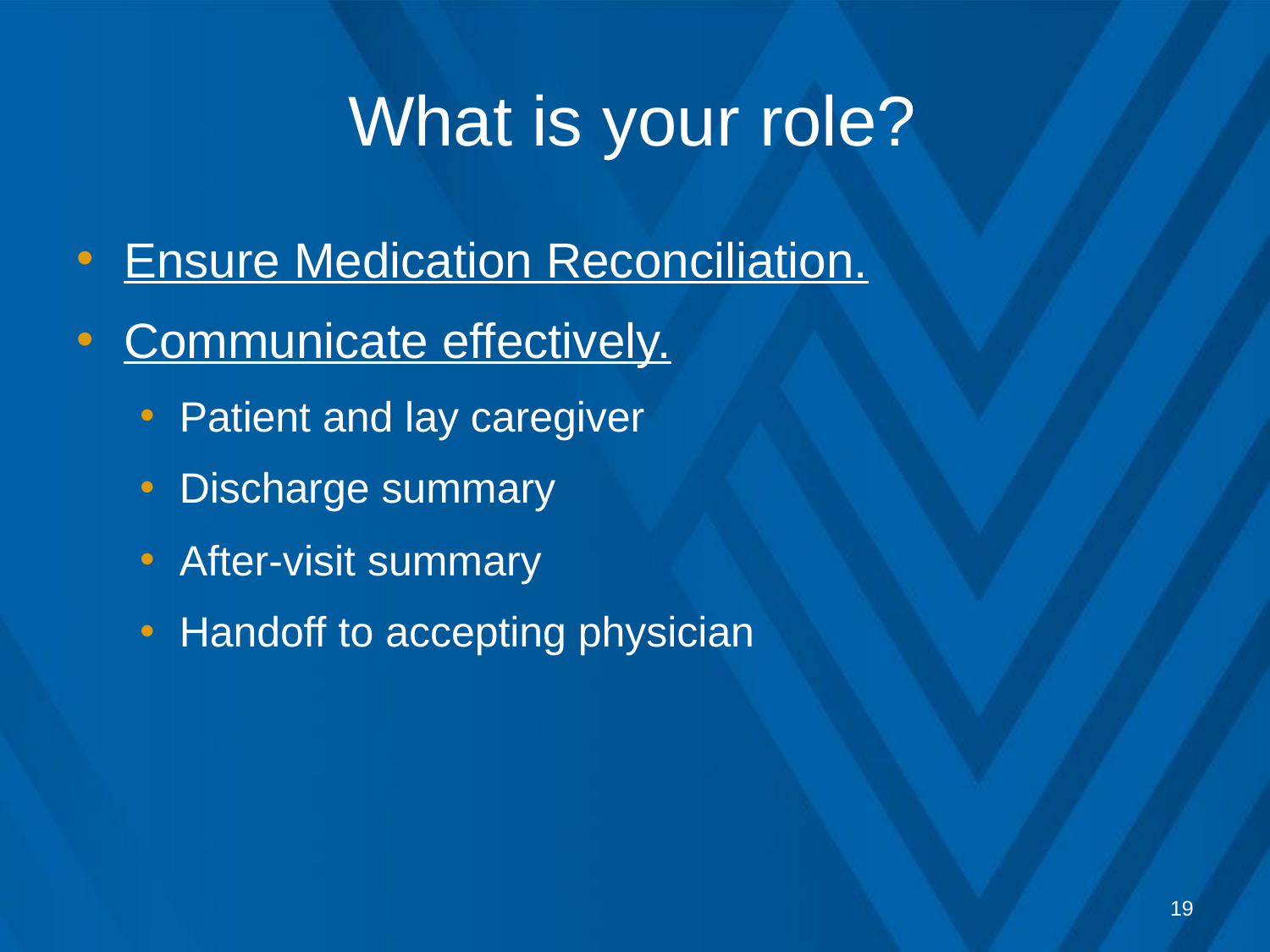

# What is your role? [2]
Ensure Medication Reconciliation.
Communicate effectively.
Patient and lay caregiver
Discharge summary
After-visit summary
Handoff to accepting physician
19

## Slide 20
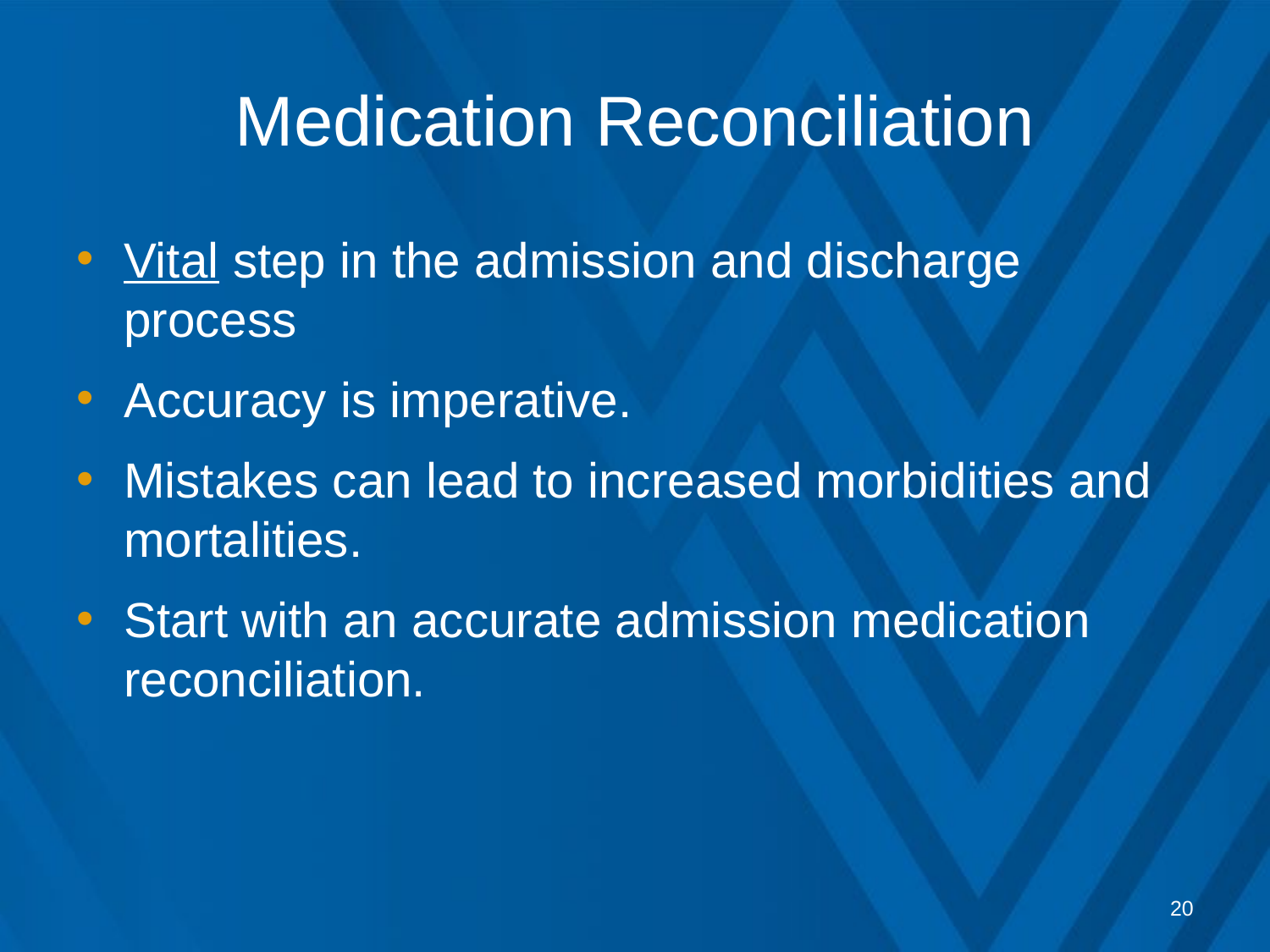

# Medication Reconciliation
Vital step in the admission and discharge process
Accuracy is imperative.
Mistakes can lead to increased morbidities and mortalities.
Start with an accurate admission medication reconciliation.
20

## Slide 21
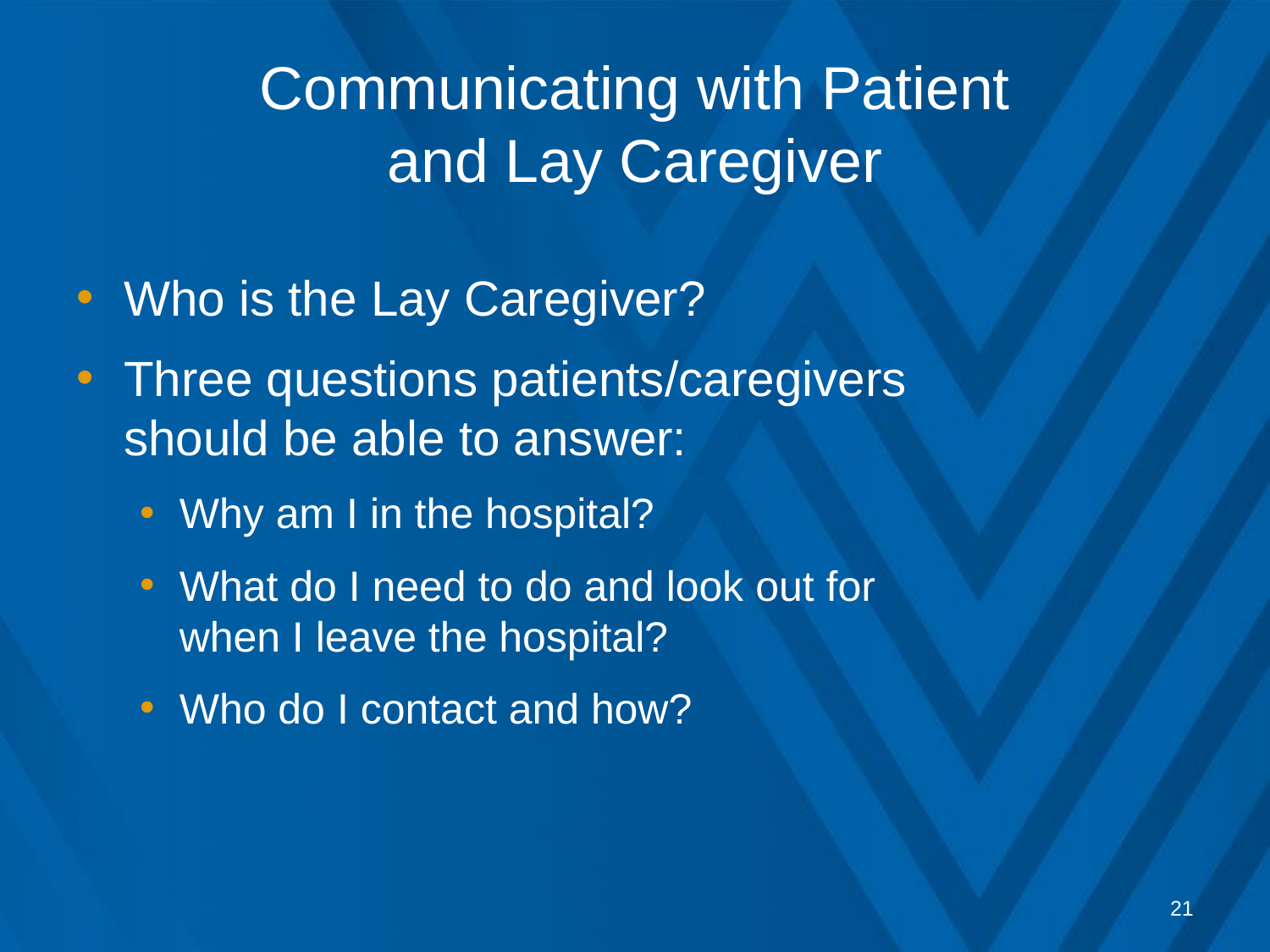

# Communicating with Patient and Lay Caregiver
Who is the Lay Caregiver?
Three questions patients/caregivers should be able to answer:
Why am I in the hospital?
What do I need to do and look out for when I leave the hospital?
Who do I contact and how?
21

## Slide 22
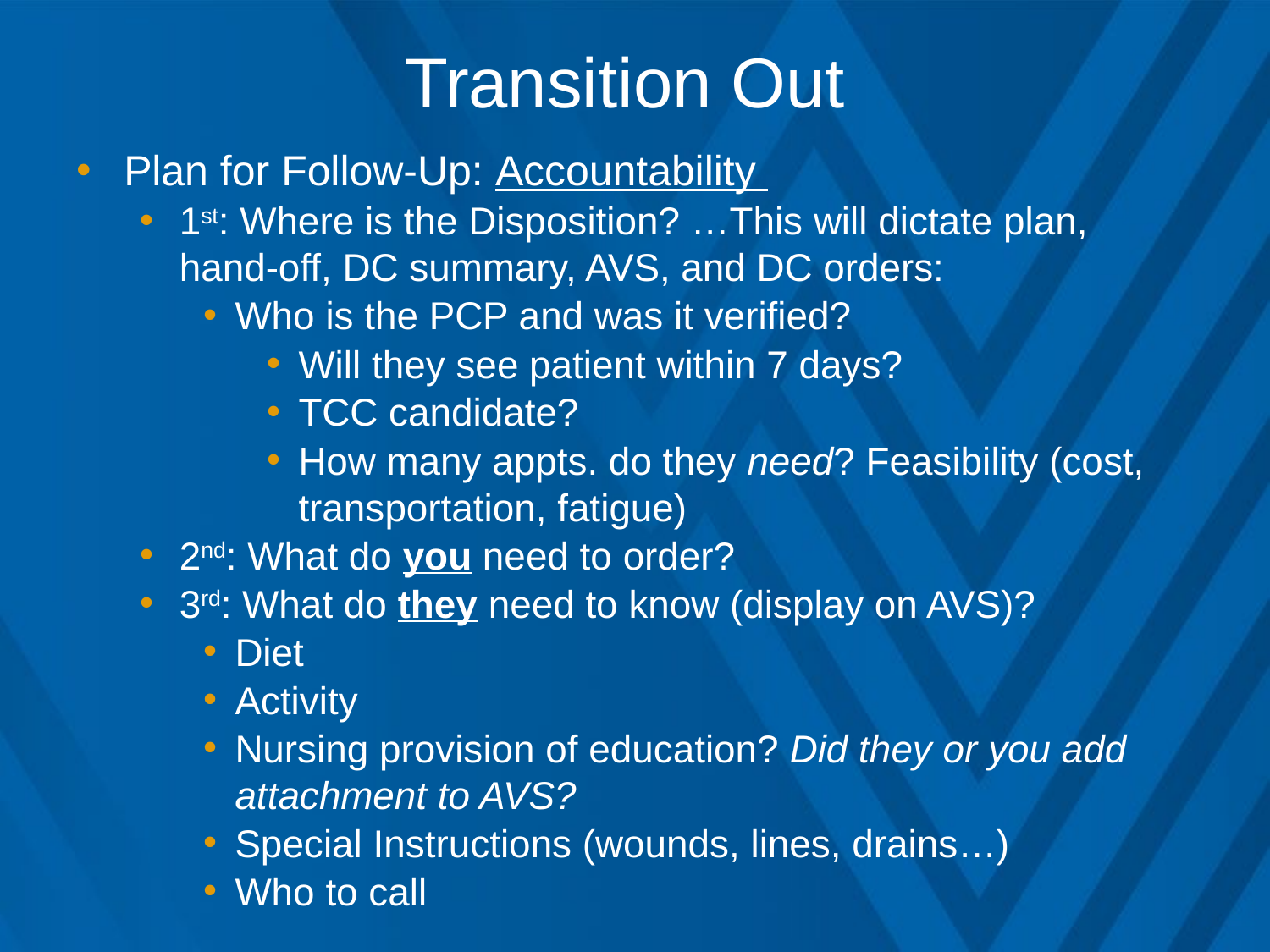

# Transition Out
Plan for Follow-Up: Accountability
1st: Where is the Disposition? …This will dictate plan, hand-off, DC summary, AVS, and DC orders:
Who is the PCP and was it verified?
Will they see patient within 7 days?
TCC candidate?
How many appts. do they need? Feasibility (cost, transportation, fatigue)
2nd: What do you need to order?
3rd: What do they need to know (display on AVS)?
Diet
Activity
Nursing provision of education? Did they or you add attachment to AVS?
Special Instructions (wounds, lines, drains…)
Who to call

## Slide 23
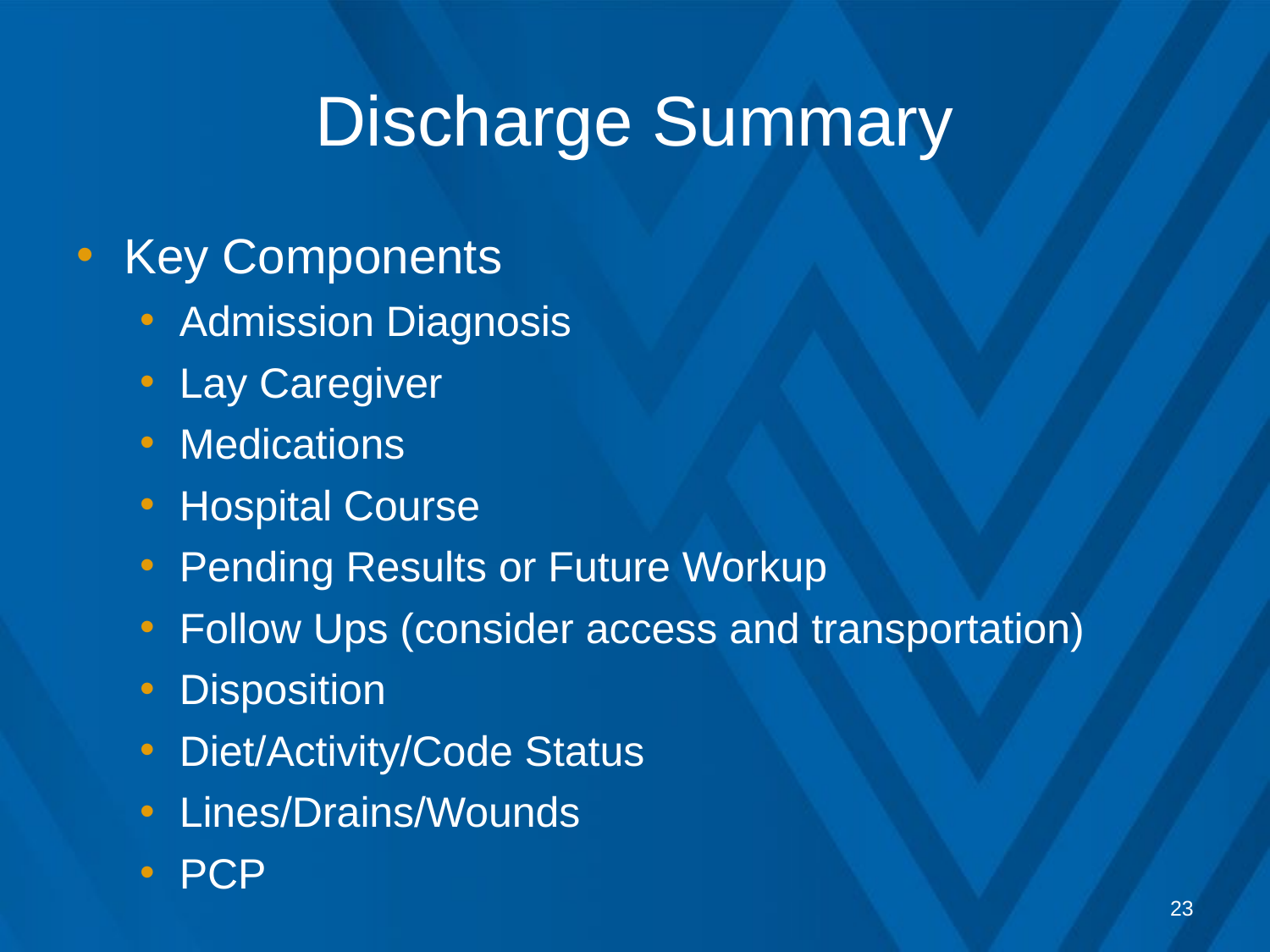

# Discharge Summary
Key Components
Admission Diagnosis
Lay Caregiver
Medications
Hospital Course
Pending Results or Future Workup
Follow Ups (consider access and transportation)
Disposition
Diet/Activity/Code Status
Lines/Drains/Wounds
PCP
23

## Slide 24
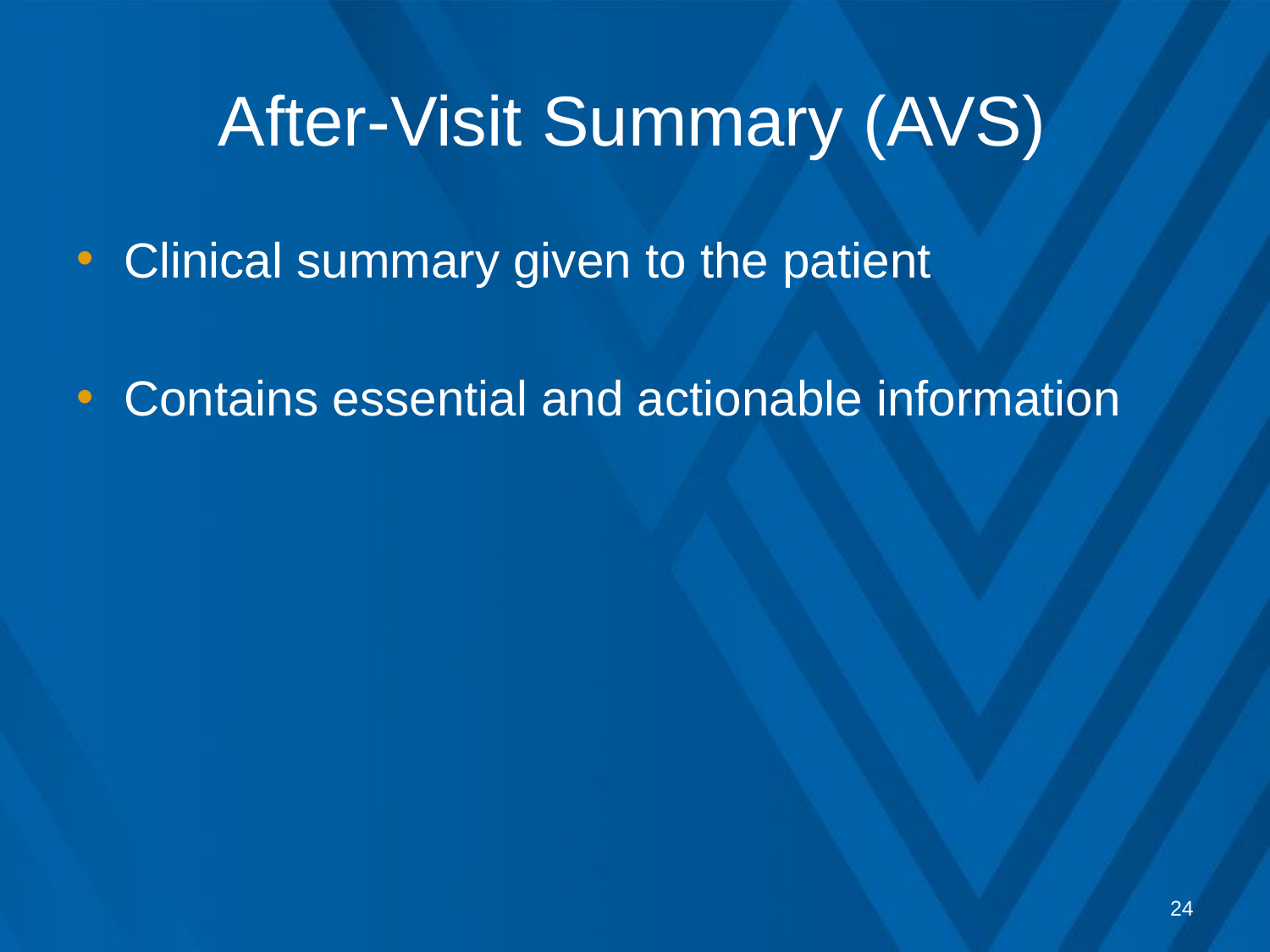

# After-Visit Summary (AVS) [1]
Clinical summary given to the patient
Contains essential and actionable information
24

## Slide 25
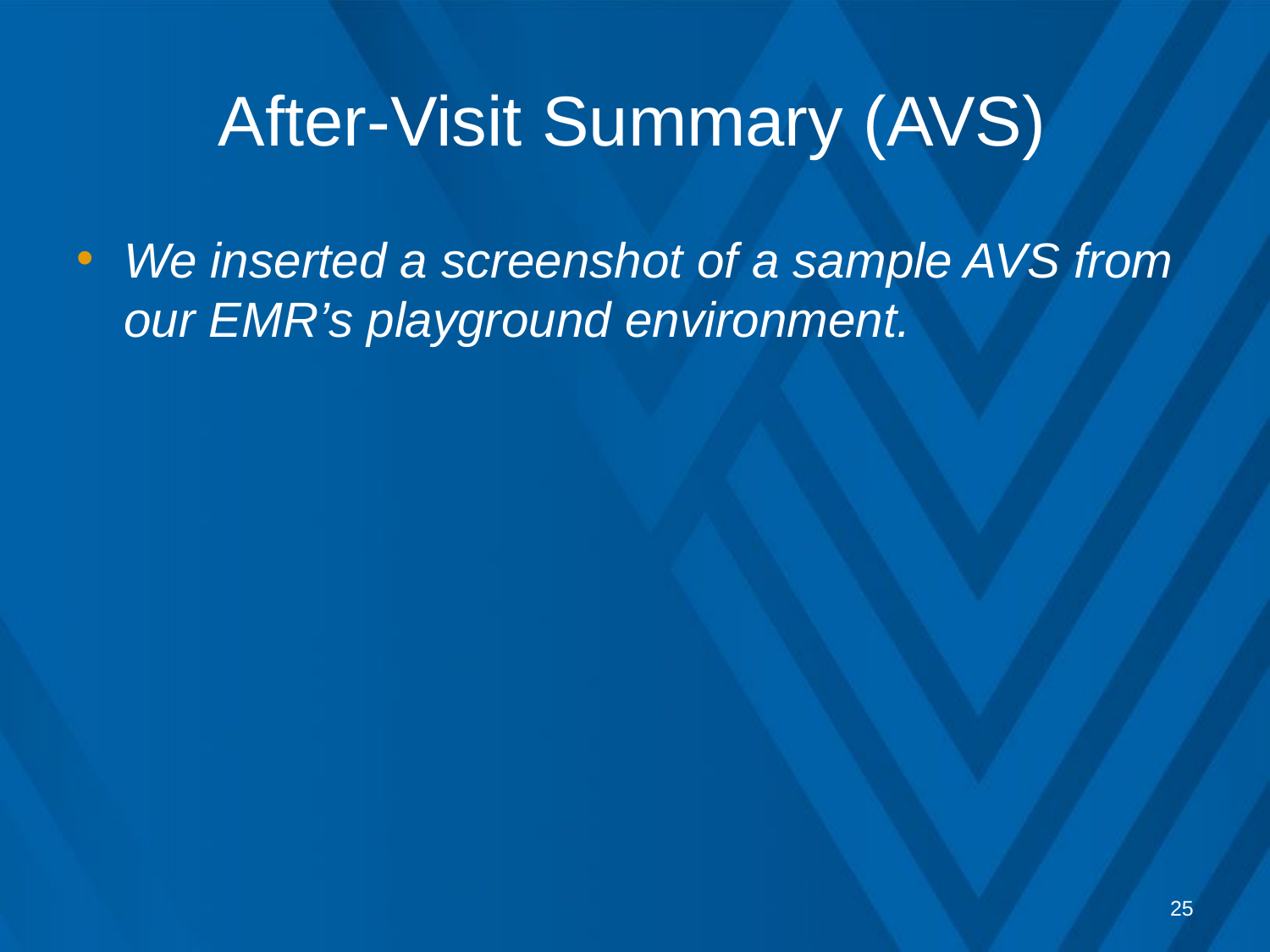

# After-Visit Summary (AVS) [2]
We inserted a screenshot of a sample AVS from our EMR’s playground environment.
25

## Slide 26
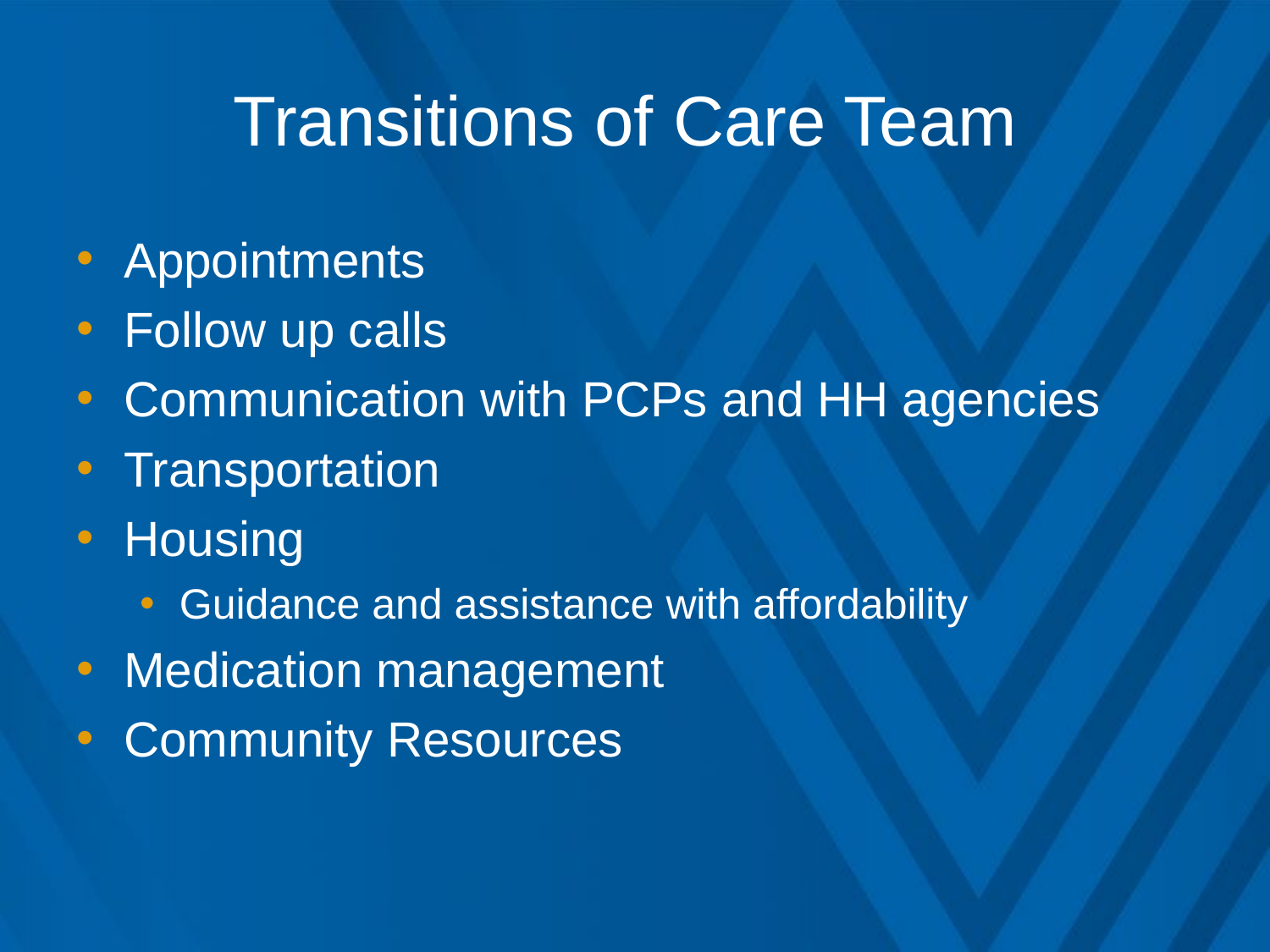

# Transitions of Care Team
Appointments
Follow up calls
Communication with PCPs and HH agencies
Transportation
Housing
Guidance and assistance with affordability
Medication management
Community Resources

## Slide 27
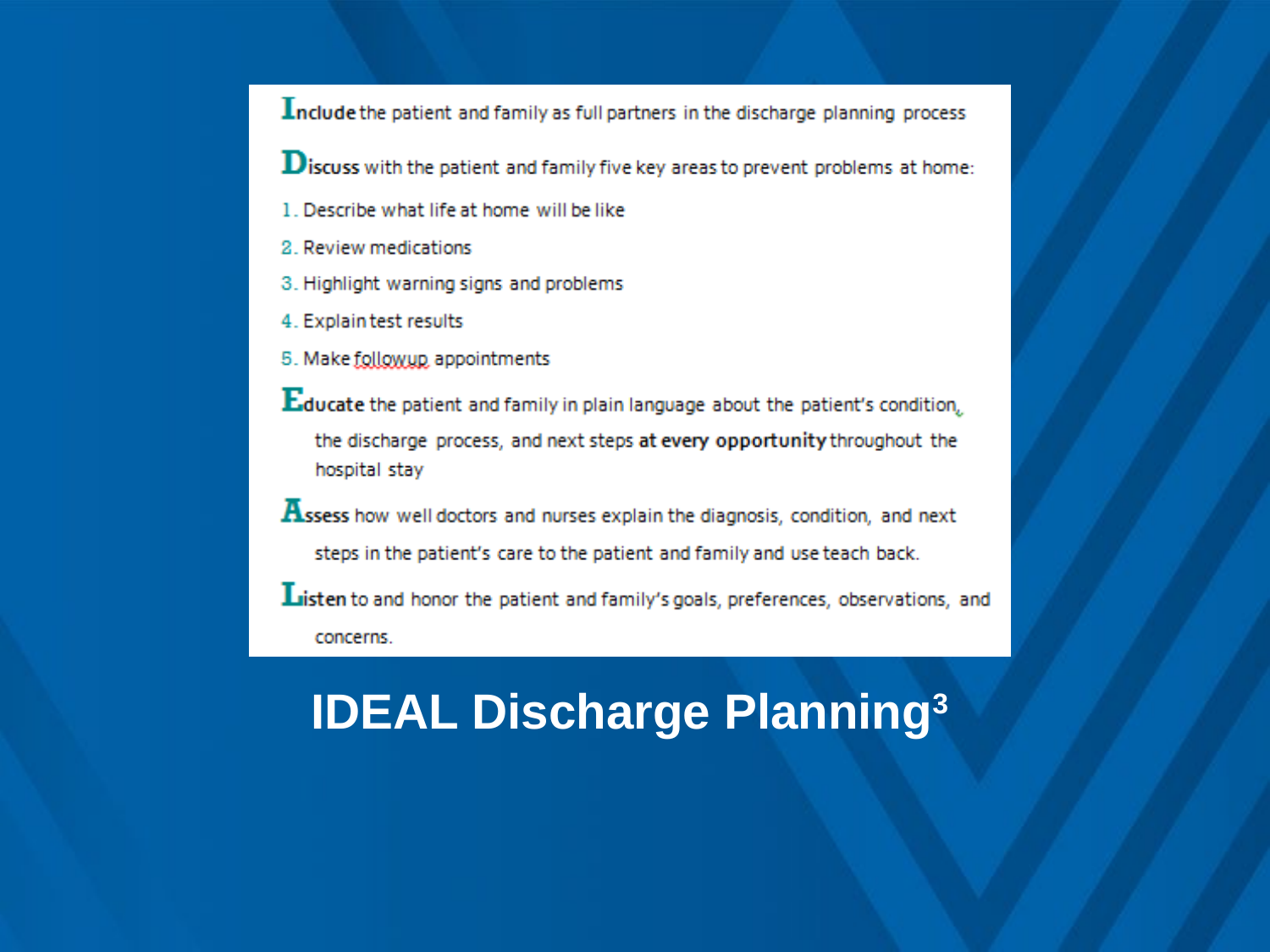

# IDEAL Discharge Planning3

## Slide 28
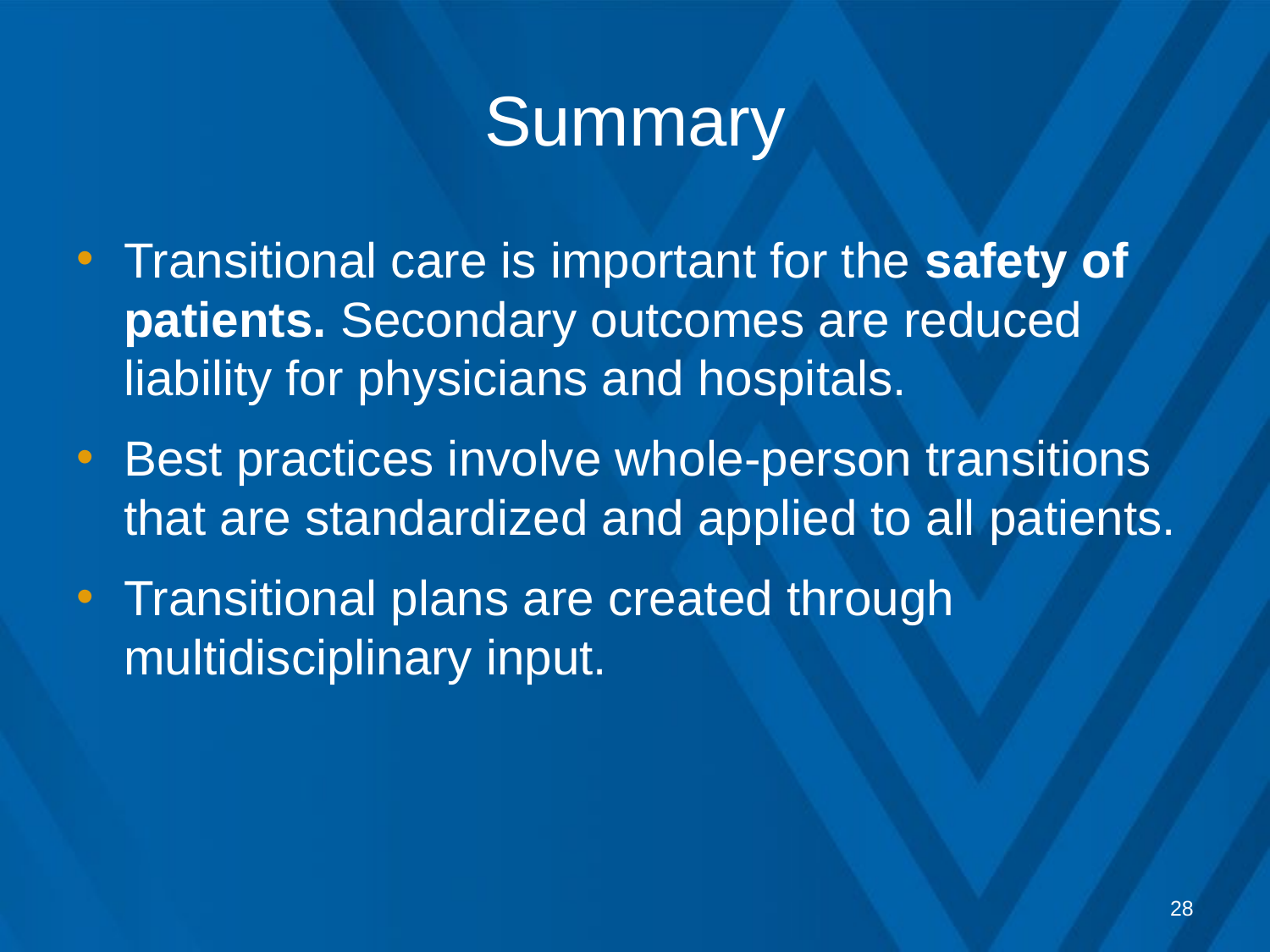

# Summary
Transitional care is important for the safety of patients. Secondary outcomes are reduced liability for physicians and hospitals.
Best practices involve whole-person transitions that are standardized and applied to all patients.
Transitional plans are created through multidisciplinary input.
28

## Slide 29
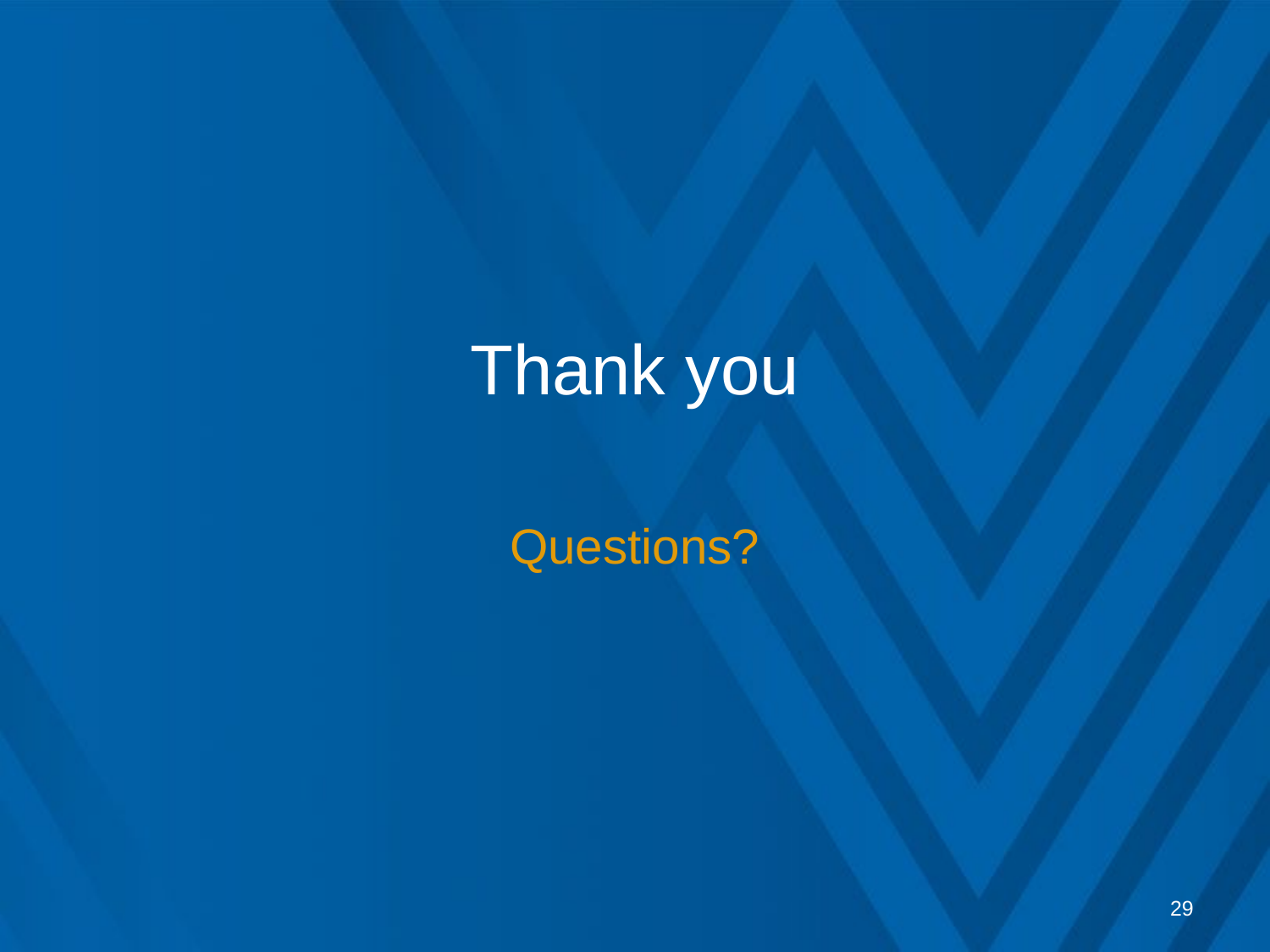

# Thank you
Questions?
29

## Slide 30
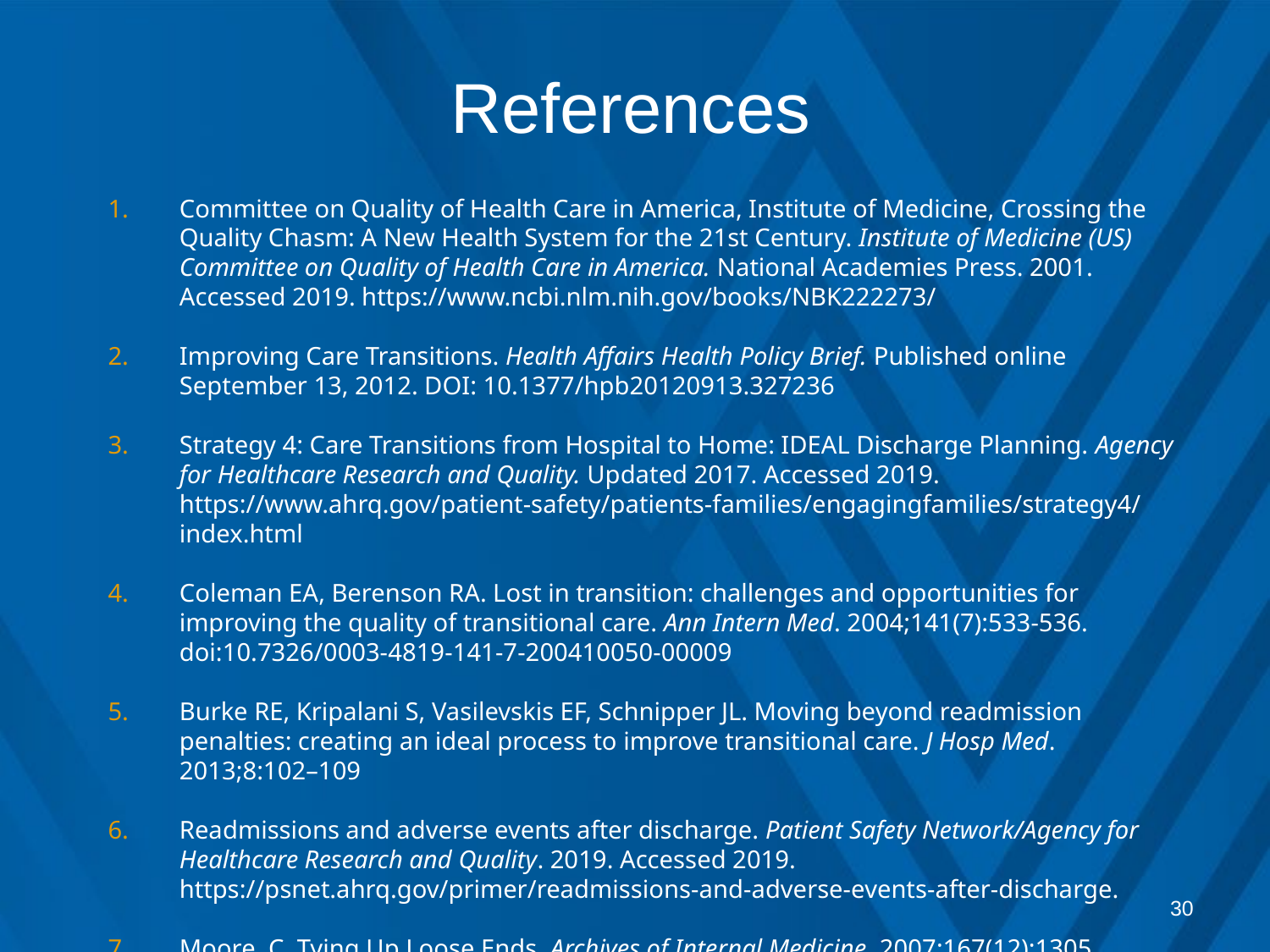

# References
Committee on Quality of Health Care in America, Institute of Medicine, Crossing the Quality Chasm: A New Health System for the 21st Century. Institute of Medicine (US) Committee on Quality of Health Care in America. National Academies Press. 2001. Accessed 2019. https://www.ncbi.nlm.nih.gov/books/NBK222273/
Improving Care Transitions. Health Affairs Health Policy Brief. Published online September 13, 2012. DOI: 10.1377/hpb20120913.327236
Strategy 4: Care Transitions from Hospital to Home: IDEAL Discharge Planning. Agency for Healthcare Research and Quality. Updated 2017. Accessed 2019. https://www.ahrq.gov/patient-safety/patients-families/engagingfamilies/strategy4/index.html
Coleman EA, Berenson RA. Lost in transition: challenges and opportunities for improving the quality of transitional care. Ann Intern Med. 2004;141(7):533-536. doi:10.7326/0003-4819-141-7-200410050-00009
Burke RE, Kripalani S, Vasilevskis EF, Schnipper JL. Moving beyond readmission penalties: creating an ideal process to improve transitional care. J Hosp Med. 2013;8:102–109
Readmissions and adverse events after discharge. Patient Safety Network/Agency for Healthcare Research and Quality. 2019. Accessed 2019. https://psnet.ahrq.gov/primer/readmissions-and-adverse-events-after-discharge.
Moore, C. Tying Up Loose Ends. Archives of Internal Medicine. 2007;167(12):1305
30

## Slide 31
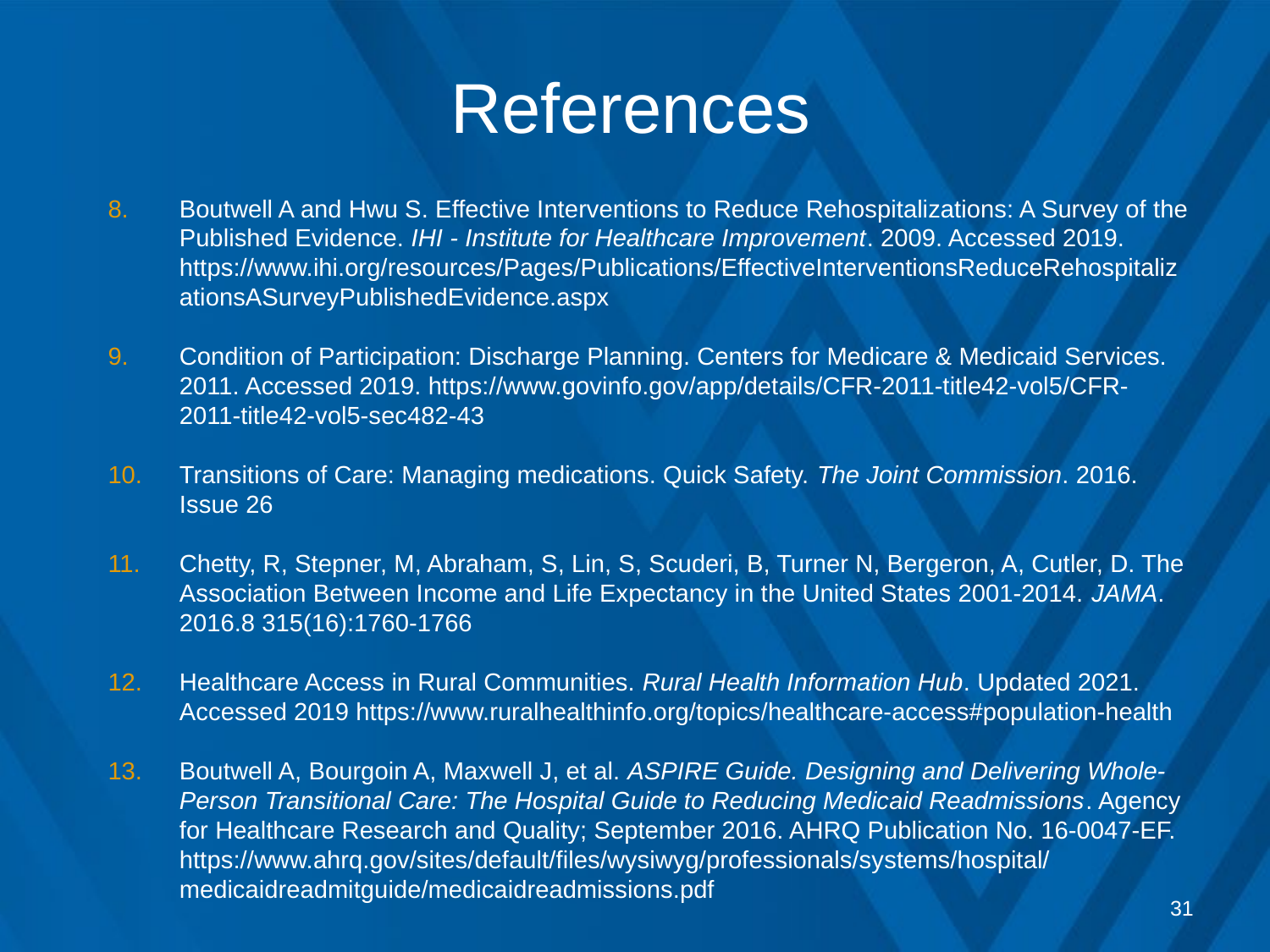

# References
Boutwell A and Hwu S. Effective Interventions to Reduce Rehospitalizations: A Survey of the Published Evidence. IHI - Institute for Healthcare Improvement. 2009. Accessed 2019. https://www.ihi.org/resources/Pages/Publications/EffectiveInterventionsReduceRehospitalizationsASurveyPublishedEvidence.aspx
Condition of Participation: Discharge Planning. Centers for Medicare & Medicaid Services. 2011. Accessed 2019. https://www.govinfo.gov/app/details/CFR-2011-title42-vol5/CFR-2011-title42-vol5-sec482-43
Transitions of Care: Managing medications. Quick Safety. The Joint Commission. 2016. Issue 26
Chetty, R, Stepner, M, Abraham, S, Lin, S, Scuderi, B, Turner N, Bergeron, A, Cutler, D. The Association Between Income and Life Expectancy in the United States 2001-2014. JAMA. 2016.8 315(16):1760-1766
Healthcare Access in Rural Communities. Rural Health Information Hub. Updated 2021. Accessed 2019 https://www.ruralhealthinfo.org/topics/healthcare-access#population-health
Boutwell A, Bourgoin A, Maxwell J, et al. ASPIRE Guide. Designing and Delivering Whole-Person Transitional Care: The Hospital Guide to Reducing Medicaid Readmissions. Agency for Healthcare Research and Quality; September 2016. AHRQ Publication No. 16-0047-EF. https://www.ahrq.gov/sites/default/files/wysiwyg/professionals/systems/hospital/medicaidreadmitguide/medicaidreadmissions.pdf
31
