# Supplementary material for: Training to Transition: Using Simulation-Based Training to Improve Resident Physician Confidence in Hospital Discharges
Source: MedEdPORTAL. 2023 Sep 15;19:11348. doi: 10.15766/mep_2374-8265.11348 (PMC10502193; doi:10.15766/mep_2374-8265.11348)
Supplement: Supplementary file 1 — Discharge Checklist Lecture.pptxPrebrief.docxSimulation Case 1.docxSimulation Case 2.docxSimulation Case Rubrics.docx [file mep_2374-8265.11348-s001.zip › B. Prebrief.docx]

Pre-Brief Case 1 and Case 2

Author: Jenna Sizemore

Welcome to your discharge simulation!

You will perform a hospital discharge on a patient that has complex medical needs and has had an extended period of time being hospitalized very far away from their home. Please reference the lecture you had recently on performing a safe discharge, especially when thinking about safely transitioning our patients out of hospitals to their home.

By the end of this activity, you will be able to:

1. Identify relevant details from a discharge summary comprised of a brief patient history, hospital course, and medication list.

2. Enter patient discharge orders in an electronic medical record using a playground computer system.

3. Communicate accurate discharge orders about medications, outpatient appointments, and outpatient laboratory investigations to a patient and caregiver based on a discharge rubric with case-specific goals.

4. Respond effectively to patient and caregiver inquiries and concerns in a simulated discharge interaction.

You will be escorted to your patient’s hospital room when you arrive to the waiting area of our simulation center. In this scenario, you just started your first day of hospital service, so this will be your first time meeting the patient. A family member will be present that is planning to assist the patient home. The patient has an anticipated discharge date of the day of your simulation encounter.

During this encounter, your goal is to discuss the discharge plan with the patient and their family member. Please review and discuss their medication list, any anticipated follow ups, or other discharge needs that you can anticipate for the patient. Please answer the questions to the best of your ability. It is normal and expected that you may not know an answer that is asked, of which you can respond that you will find the answer for the patient. You will be allotted roughly 30 minutes to discuss the discharge plan with the patient and their family member, after which we will allow 10 minutes of dedicated feedback. Please feel free to ask any questions during this time as well. A facilitator will be present to observe the scenario as well. This is a low stakes encounter designed to help you become more comfortable with discharges.

After determining a discharge plan of care with the patient, you will leave the room and place discharge orders in the discharge navigator in a playground EMR environment. A physician facilitator will be present to supervise your order entry, as well as assist or answer any questions that you may have. You will have roughly 15 minutes to complete your discharge order reconciliation.

A brief hospital summary follows. You will be notified if you are assigned to Case 1 or Case 2.

Case 1:

Patient Name: Jo Smith, Adult Patient

Hospital Service: Medicine 4

Discharge Diagnosis: CAD, DM II

Admitting diagnosis: DKA, NSTEMI II

Hospital Problem List:

NSTEMI (resolved)

Diabetes Mellitus, Type II

DKA (resolved)

HTN

HLD

Discharge Medication List:

Glargine sub q injection, 45 units qhs

Aspart sub q injection, 5 units pre-meal

Aspirin 81 mg qd

Ticagrelor 90 mg bid

Lisinopril 2.5 mg qd

Metoprolol tartrate 25 mg bid

Atorvastatin 80 mg daily

Brief Hospital Course: Adult patient admitted to a large academic hospital with an acute non ST elevation myocardial infarction and diabetic ketoacidosis. The patient received a left heart catheterization, with access through the right groin, with deployment of 2 drug eluting stents to the LAD and RCA. She was started on insulin and fluids for glucose control which resolved the diabetic ketoacidosis. The patient has continued on insulin injections, both basal and pre-prandial, throughout the hospitalization, though it did require several days to achieve adequate control of blood glucose. The patient was started on dual antiplatelet therapy with Ticagrelor and Aspirin, as well as atorvastatin, metoprolol tartrate, and Lisinopril. Overall, the patient feels much improved. The left heart catheterization was tolerated well without the development of any pseudoanuerysm or groin hematoma.

Activity/ambulation: Cleared for full activity without restrictions by Cardiology. No ambulatory restrictions.

DMC: Full capacity

Diet: Diabetic

Case 2:

Patient Name: Jo Smith, Adult patient

Hospital Service: Medicine 4

Admitting Diagnosis: Sepsis, MRSA bacteremia

Discharge Diagnosis: OUD, MRSA Aortic Valve Endocarditis s/p mechanical valve replacement, Mood Disorder, need for lifelong anticoagulation

Hospital Problem List:

Opioid Use disorder, severe, in early remission

MRSA Endocarditis and Bacteremia

Sepsis, resolved.

Aortic Mechanical Valve replacement

Coumadin therapy monitoring

Major Depressive Disorder

Generalized Anxiety Disorder

Discharge Medication List:

Warfarin 2.5 mg Mon, Wed, Fri, 3 mg Tues, Thur, Sat

Sertraline 75 mg daily

Buprenorphine-Naloxone 8 mg/2 mg daily

Buspirone 30 mg daily

Brief Hospital Course: Adult patient admitted with *Methicillin-resistant staph aureus* with recent exposure to injected opioids was initially received as a transfer from a rural, critical access hospital several weeks ago. The patient was ultimately diagnosed with aortic valve endocarditis with embolic phenomenon, including CNS lesions. The patient received a successful mechanical valve replacement 5 weeks prior to discharge. After clearance from cardiac surgery, the patient was admitted to the multidisciplinary infusion service. Infectious disease and Psychiatry were consulted and have been actively following the patient. The patient completed 6 weeks of therapy with IV Vancomycin, started on buprenorphine-naloxone, and is currently in clinical remission for opioid use disorder. The patient underwent intensive cognitive behavioral therapy while admitted and completed a 6 weeks course of IV antibiotics. Anticoagulation was initiated with warfarin for the mechanical aortic valve. INR at time of discharge is 2.3. Laboratory monitoring remained stable for the last three weeks of hospitalization.

Activity/ambulation: Cleared for full activity without restrictions by CT surgery. No ambulatory restrictions.

DMC: Full capacity

Diet: Regular
